# Exploring the genetic landscape of ciprofloxacin-induced DNA supercompaction in *Escherichia coli*

**DOI:** 10.1093/nar/gkag573

**Published:** 2026-06-22

**Authors:** Krister Vikedal, Natalia Berges, Ida Mathilde Marstein Riisnæs, Synnøve Brandt Ræder, Jørgen Vildershøj Bjørnholt, Magnar Bjørås, Kirsten Skarstad, Emily Helgesen, James Alexander Booth

**Affiliations:** Department of Microbiology, Oslo University Hospital, Rikshospitalet, 0373 Oslo, Norway; Department of Microbiology, University of Oslo, 0316 Oslo, Norway; Department of Microbiology, Oslo University Hospital, Rikshospitalet, 0373 Oslo, Norway; Department of Microbiology, Oslo University Hospital, Rikshospitalet, 0373 Oslo, Norway; Department of Microbiology, University of Oslo, 0316 Oslo, Norway; Department of Microbiology, Oslo University Hospital, Rikshospitalet, 0373 Oslo, Norway; Department of Microbiology, Oslo University Hospital, Rikshospitalet, 0373 Oslo, Norway; Department of Microbiology, University of Oslo, 0316 Oslo, Norway; Department of Microbiology, Oslo University Hospital, Rikshospitalet, 0373 Oslo, Norway; Department of Microbiology, University of Oslo, 0316 Oslo, Norway; Department of Clinical and Molecular Medicine, Norwegian University of Science and Technology, 7030 Trondheim, Norway; Department of Microbiology, Oslo University Hospital, Rikshospitalet, 0373 Oslo, Norway; Department of Microbiology, Oslo University Hospital, Rikshospitalet, 0373 Oslo, Norway; Department of Clinical and Molecular Medicine, Norwegian University of Science and Technology, 7030 Trondheim, Norway; Department of Microbiology, Oslo University Hospital, Rikshospitalet, 0373 Oslo, Norway; Department of Clinical and Molecular Medicine, Norwegian University of Science and Technology, 7030 Trondheim, Norway

## Abstract

DNA-damaging antibiotics like ciprofloxacin (CIP) induce extensive double-strand breaks in *Escherichia coli*, triggering both the SOS response and rapid DNA supercompaction. To uncover genes involved in the latter process beyond the previously identified key orchestrators encoded by *recN* and *recA*, we developed a novel machine learning-assisted high-throughput screening workflow and applied it to nearly 4000 *E. coli* strains, including the Keio collection’s single-gene deletion strains and additional in-house strains. Conservative validation identified 15 hit strains with impaired DNA supercompaction. While defects in recombinational repair genes were associated with the most severe impairments, our investigation also revealed genes not previously associated with DNA compaction or repair that had milder and more heterogeneous effects on supercompaction, including *yaiW*, which encodes a membrane-associated protein. Notably, several non-DNA-repair gene deletions affected RecN colocalization with the nucleoid, *recN* expression, SOS response activity, or survival after CIP exposure, supporting indirect or modulatory roles. Altogether, this work confirms RecN and RecA as primary drivers of DNA supercompaction and demonstrates that high-content imaging combined with machine learning-assisted analysis provides a scalable approach to explore bacterial DNA organization phenotypes and DNA damage responses.

## Graphical Abstract

**Graphical Abstract:**
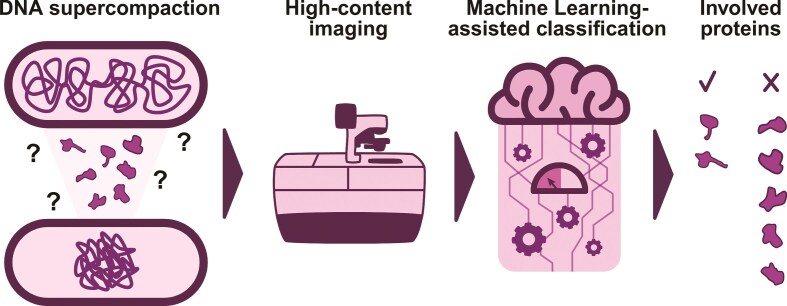

## Introduction

The emergence and spread of fluoroquinolone antibiotic resistance represents a critical challenge to global health [1]. Ciprofloxacin (CIP), a widely prescribed fluoroquinolone, damages DNA by forming adducts with DNA gyrase and Topoisomerase IV, thereby blocking the progression of the replication machinery [2–4]. These effects ultimately result in the formation of DNA double-strand breaks (DSBs) and trigger the major bacterial DNA damage response known as the SOS response [2]. When there is no DNA damage, the transcription factor LexA binds to regions upstream of DNA repair genes, thereby repressing their transcription. Upon DNA damage, RecA binds to single-stranded DNA (ssDNA), where it not only participates in DNA repair but also triggers the SOS response by promoting the autocleavage of the LexA repressor [5, 6].

The SOS response constitutes a sophisticated cellular program that coordinates multiple survival mechanisms [7]. Upon activation, this system triggers extensive changes in gene expression, leading to the production of DNA repair proteins, protective factors, and a multitude of uncharacterized gene products [8, 9]. For instance, the SOS response induces the expression of proteins necessary for complex DSB repair mechanisms centered around homologous recombination [10]. This process begins when the RecBCD complex processes DNA ends at DSBs, generating ssDNA that serves as a substrate for RecA protein loading [11]. RecA then catalyzes the search for and pairing with intact homologous sequences, enabling repair of the damaged DNA region [12, 13].

The SMC-like protein RecN has also been established as an important factor in DSB repair [14, 15] and stands out as one of the most highly expressed proteins during the SOS response [8, 9]. Its protein levels are tightly controlled through rapid degradation by the ClpXP protease system [16, 17]. Although RecN-deficient cells show increased sensitivity to CIP-induced DNA damage [18], the precise mechanism by which RecN operates and contributes to DSB repair remains incompletely understood.

A striking aspect of the bacterial response to DNA damage is nucleoid compaction, observed following exposure to various genotoxic agents including ultraviolet (UV) irradiation, mitomycin C, bleomycin, and fluoroquinolones such as CIP and ofloxacin [18–22]. Recently, we discovered that severe DNA damage induced by agents like CIP triggers a stepwise reorganization of the nucleoid, a process we termed “DNA supercompaction” [18]. Upon severe DNA damage, the cell’s nucleoid rapidly transitions from its multifocal distribution—common in unchallenged conditions—through quarter-position compaction, culminating in a dense, persistent midcell compaction. Quarter-position compaction involves the formation of two distinct nucleoid lobes situated in separate cell halves. Although the DNA supercompaction process depends on both RecA and RecN, a quarter-position compaction occurs even in their absence, albeit in a temporally dysregulated manner [18]. The apparent independence of quarter-position compaction from RecA and RecN suggests that additional, unidentified genetic factors may influence the DNA supercompaction process.

The Keio collection of *Escherichia coli* strains, featuring single-gene deletions of all nonessential genes from the BW25113 background [23–25], has been widely used to explore various bacterial phenomena [26–31]. In this study, we aimed to explore the broader genetic landscape of CIP-induced DNA supercompaction and establish a novel machine learning-assisted high-throughput screening workflow for identifying strains with altered supercompaction phenotypes. Using high-content imaging, machine learning-assisted phenotype classification, and a conservative validation process, we systematically screened the Keio collection together with additional in-house strains. This workflow identified 15 hit strains with impaired DNA supercompaction, deviating from wild-type-like midcell compaction. These strains featured defects in both known recombinational repair genes and genes not previously associated with DNA compaction or repair, with the former leading to more severe impairments. Follow-up analyses of the non-DNA-repair hits associated their milder impairments with heterogeneous effects on RecN dynamics, *recN* expression, and survival after CIP exposure.

## Materials and methods

### Strains used in screening and growth conditions

For the screening, we included a total of 3862 *E. coli* strains, comprising 3797 nonessential single-gene deletion strains from the Keio collection [23] and 65 strains from our in-house library (Supplementary Table S1). The in-house strains, featuring various genetic backgrounds with single or multiple gene mutations and deletions, were included due to their potential relevance to the DNA supercompaction process. To facilitate high-throughput handling, strains were distributed across a series of 13 different 384-well plates (Merck Life Science no. P5991), excluding outer wells. Each plate contained two samples of a wild-type strain (BW25113) [32] and a *ΔrecN* strain (JW5416) [23] as controls for the DNA supercompaction process [18].

Strains were cultured in LB medium with relevant antibiotics (30 µl per well) within 384-well plates and incubated at 37°C in sealed bags with high humidity to prevent drying., Kanamycin (30 µg/ml) was used for Keio collection strains, while for the in-house strains, antibiotics as specified in Supplementary Table S1 were used at the following concentrations: 20 µg/ml chloramphenicol, 5 µg/ml tetracycline, and 50 µg/ml ampicillin.

### Sample preparation for screening

Overnight cultures (ONCs) were inoculated directly from freeze stock using a 96-pin Multi-Blot Replicator (V & P Scientific no. VP 408FP6 and VP381). On the day of imaging, new cultures were inoculated from the ONCs and incubated until the OD_600_ of control samples reached 0.4. Next, CIP (10 µg/ml) was added to all wells, excluding one sample for each of the control strains, to induce severe DNA damage and trigger a rapid, ordered DNA supercompaction response [18]. Samples were incubated with CIP for 15–20 min before fixation with ethanol (50% final concentration).

For imaging, glass-bottomed 384-well plates (Fisher Scientific no. 10687354) were pre-treated with 25 µl Poly-L-Lysine (Merck Life Science no. P8920) for 8 min and washed once with phosphate buffered saline (PBS). Fixed samples (15 µl) were transferred to these plates and DNA was stained with 5 µg/ml Hoechst 33258. During staining, plates were centrifuged at 700 × *g* for 10 min to ensure sufficient cell adhesion to the bottom of the well. After centrifugation, the supernatant was carefully removed, wells were washed once with PBS, before finally adding 25 µl PBS. Plates with samples were brought to the microscope for imaging immediately after preparation and maintained at room temperature throughout the procedure.

### High-content imaging for screening

Samples in glass-bottomed 384-well plates were imaged using an ImageXpress Micro Confocal High-Content Imaging System (Molecular Devices), equipped with a 60× air objective (Nikon CFI Plan Apochromat Lambda 60XC, 0.95 NA) and an Andor Zyla sCMOS camera. Imaging and focusing were fully automatic, though each plate’s automatic focus setup was verified and adjusted as needed. Four locations per well were imaged, spaced evenly by 600 µm, using two channels with auto-shading correction: a transmitted light (TL) channel for detecting cell outlines and a fluorescence channel for Hoechst 33 258, with excitation at 350–404 nm and emission collection at 417–477 nm. The optimal focus for each location was automatically selected from a Z-stack of three images separated by 0.4 µm in each direction from the default focus position. Edge wells were excluded from imaging due to interference between the objective and the plate skirt (according to microscope manual), a limitation accounted for during strain plate preparation.

### Image analysis and strain classification in screening

We utilized the open-source software CellProfiler (version 4) [33] to preprocess and analyze images from the screening, together with its companion tool CellProfiler Analyst (version 3) [34] for training machine learning models for cell classification. Full pipelines are available in our Zenodo repository (see the ‘Data availability’ section). All images, with a pixel size of 115 nm × 115 nm, were preprocessed to remove background noise. The TL channel was enhanced to isolate dark hole elements 7–35 pixels in diameter and suppressed for elements smaller than 6 pixels wide. The fluorescence channel underwent enhancement for speckle elements up to 18 pixels in diameter. Cells were segmented from these preprocessed TL channels by identifying objects with diameters of 8–35 pixels, using a global “Robust Background” thresholding method with mode averaging. Cell segmentation did not involve splitting of cell clusters. DNA foci were identified from corresponding fluorescence channels by detecting objects smaller than 35 pixels in diameter, employing a global “Robust Background” thresholding method with median averaging. Clustered DNA foci were distinguished by separating local intensity peaks at least 4 pixels apart after application of a smoothing filter. Holes in identified DNA objects were filled, and only cell objects collocating with DNA foci were included in subsequent analyses. A range of measurements were collected for the segmented cell objects and their DNA foci, encompassing features such as size, shape, and DNA distribution (see Supplementary Material).

Two machine learning models were developed for cell classification using CellProfiler Analyst: the Single-Cell model and the Phenotype model. These models were trained using a 15-parameter FastGentleBoosting classification strategy within CellProfiler Analyst, utilizing both simple and complex measurements from CellProfiler analyses (training details in Supplementary Material). The Single-Cell model refined cell segmentation accuracy by excluding clusters, imaging artefacts, and out-of-focus cells, establishing a solid basis for subsequent phenotype classifications. The Phenotype model classified cells by their DNA supercompaction phenotypes—defined in this study as wild-type, *ΔrecN*, and unchallenged. This classification provided insights into phenotype prevalence across samples and was fundamental for enrichment scoring and candidate strain identification.

To efficiently process the large volume of images generated in the screening, we employed CellProfiler’s batch processing capabilities on cluster computers to automate image analysis. This approach generated extensive spreadsheets with measurements for various features of every individual cell. We developed a Python script to compile these results from all cells within a given sample, conduct statistical analyses, and organize the data into a manageable format for further review. Enrichment scores for DNA supercompaction phenotypes were calculated for every sample (see Supplementary Material) using the same method employed by CellProfiler Analyst [35]. All relevant scripts are available in our Zenodo repository (see the ‘Data availability’ section).

### Growth conditions for individual culturing

Strains were cultured individually to ensure they reached exponential growth prior to treatments and fixation. Initially, frozen stocks were streaked onto LB-agar plates and incubated overnight at 37°C. The following day, fresh growth medium was inoculated with 5–10 colonies from each strain and incubated overnight with shaking. At the start of an experiment, ONCs were diluted depending on cell density (1:500 or 1:200 for most) in fresh growth medium and cultured to exponential growth (OD_600_ around 0.2–0.4) before applying treatments. LB medium was generally used for culturing, except for in a screening validation experiment where AB minimal medium [36] supplemented with 0.5% casamino acids, 0.2% glucose, and 1 µg/ml thiamine was utilized to provide a different growth condition [37]. Cells were exposed to CIP at a dose of 10 µg/ml for 20 min, unless otherwise specified, to effectively differentiate the compaction phenotypes among strains [18]. Ethanol at a 50% final concentration was used for cell fixation. Keio collection strains were cultured with 30 µg/ml Kanamycin, while in-house and constructed strains were cultured using antibiotics as indicated in Table 1 and Supplementary Table S1, at the following concentrations: 100 µg/ml ampicillin, 20 µg/ml chloramphenicol, 30 µg/ml kanamycin, and 5 µg/ml tetracycline.

**Table 1.** Strains used in this study to investigate the role of hit strain mutations and deletions in DNA supercompaction

Strain	Relevant genotype	Source
BW25113	Wild type	[32]; CGSC7636
JW5416	BW25113 *ΔrecN::kan* [Kan^R^]	[23]; Keio collection
AB1157	Wild type	[46, 47]; Lab collection
BL21	Wild type	[48, 49]; Lab collection
CCUG17620	Wild type	[50]; corresponds to ATCC25922
MG1655	Wild type	[51]; CGSC7740
KV52	BW25113 *recA306*^a^::*Tn10* [Tet^R^]	This work; P1 STL9253 × BW25113
SBR14	MG1655 *ΔrecA::kan* [Kan^R^]	This work; P1 JW2669 × MG1655 [23]
EH75	AB1157 *ΔrecA::kan* [Kan^R^]	Lab collection; P1 JW2669 × AB1157 [23]
SS6282	MG1655 *hupA100::mCherry::kan ΔattB::psulA-gfp* [Kan^R^]	[52]; S. Sandler
KV21	BW25113 *hupA100::mCherry*	[18]
KV60	BW25113 *hupA100::mCherry* + pSG101 (pSOS) [Amp^R^]	[18]
MR16	BW25113 *ΔrecN hupA100::mCherry* [Kan^R^]	[18]
MR23	BW25113 *ΔclpS hupA100::mCherry::kan* [Kan^R^]	This work; P1 SS6282 × JW0865 (*kan* removed with pCP20)
MR24	BW25113 *ΔdusB hupA100::mCherry::kan* [Kan^R^]	This work; P1 SS6282 × JW3228 (*kan* removed with pCP20)
MR25	BW25113 *ΔyaiW hupA100::mCherry::kan* [Kan^R^]	This work; P1 SS6282 × JW0369 (*kan* removed with pCP20)
MR26	BW25113 *Δhfq hupA100::mCherry::kan* [Kan^R^]	This work; P1 SS6282 × JW4130 (*kan* removed with pCP20)
MR27	BW25113 *ΔydeE hupA100::mCherry::kan* [Kan^R^]	This work; P1 SS6282 × JW1527 (*kan* removed with pCP20)
MR28	SMG3^b^*hda*_F85V_*hupA100::mCherry::kan* [Tet^R^, Kan^R^]	This work; P1 SS6282 × KS1115
MR29	BW25113 *ΔrecB hupA100::mCherry::kan* [Kan^R^]	This work; P1 SS6282 × JW2788 (*kan* removed with pCP20)
MR30	BW25113 *ΔrecC hupA100::mCherry::kan* [Kan^R^]	This work; P1 SS6282 × JW2790 (*kan* removed with pCP20)
MR31	BW25113 *ΔrecD hupA100::mCherry::kan* [Kan^R^]	This work; P1 SS6282 × JW2787 (*kan* removed with pCP20)
MR32	BW25113 *ΔrecF hupA100::mCherry::kan* [Kan^R^]	This work; P1 SS6282 × JW3677 (*kan* removed with pCP20)
MR33	BW25113 *ΔrecR hupA100::mCherry::kan* [Kan^R^]	This work; P1 SS6282 × JW0461 (*kan* removed with pCP20)
KV99	BW25113 *recA306*^a^::*Tn10 hupA100::mCherry::kan* [Tet^R^, Kan^R^]	This work; P1 STL9253 × KV21; [43–45]
KV100	BW25113 *recA938::cat hupA100::mCherry::kan* [Cam^R^, Kan^R^]	This work; P1 ALS972 × KV21; [53]
KV82	MR23 (*ΔclpS*) + pSG101 (pSOS) [Kan^R^, Amp^R^]	This work
KV83	MR24 (*ΔdusB*) + pSG101 (pSOS) [Kan^R^, Amp^R^]	This work
KV84	MR25 (*ΔyaiW*) + pSG101 (pSOS) [Kan^R^, Amp^R^]	This work
KV85	MR26 (*Δhfq*) + pSG101 (pSOS) [Kan^R^, Amp^R^]	This work
KV86	MR27 (*ΔydeE*) + pSG101 (pSOS) [Kan^R^, Amp^R^]	This work
KV101	MR28 (*hda*_F85V_) + pSG101 (pSOS) [Tet^R^, Amp^R^]	This work
KV39	JW0865 (*ΔclpS*) + p*lexA*-*gfp* [Kan^R^, Cam^R^]	This work
KV40	JW3228 (*ΔdusB*) + p*lexA*-*gfp* [Kan^R^, Cam^R^]	This work
KV41	JW1527 (*ΔydeE*) + p*lexA*-*gfp* [Kan^R^, Cam^R^]	This work
KV42	JW2669 (*ΔrecA*) + p*lexA*-*gfp* [Kan^R^, Cam^R^]	This work
KV43	BW25113 (Wild type) + p*lexA*-*gfp* [Cam^R^]	This work
KV44	KS1115 (*hda*_F85V_) + p*lexA*-*gfp* [Tet^R^, Cam^R^]	This work
KV47	JW0369 (*ΔyaiW*) + p*lexA*-*gfp* [Kan^R^, Cam^R^]	This work
KV48	JW4130 (*Δhfq*) + p*lexA*-*gfp* [Kan^R^, Cam^R^]	This work
KV102	STL9253 (*recA306^a^*) + p*lexA*-*gfp* [Tet^R^, Cam^R^]	This work; [43–45]
KV103	ALS972 (*recA938*) + p*lexA*-*gfp* [Cam^R^, Kan^R^]	This work; [53]
KV104	JW2788 (*ΔrecB*) + p*lexA*-*gfp* [Kan^R^, Cam^R^]	This work
KV105	JW2790 (*ΔrecC*) + p*lexA*-*gfp* [Kan^R^, Cam^R^]	This work
KV106	JW2787 (*ΔrecD*) + p*lexA*-*gfp* [Kan^R^, Cam^R^]	This work
KV107	JW3677 (*ΔrecF*) + p*lexA*-*gfp* [Kan^R^, Cam^R^]	This work
KV108	JW0461 (*ΔrecR*) + p*lexA*-*gfp* [Kan^R^, Cam^R^]	This work
KV109	JW5416 (*ΔrecN*) + p*lexA*-*gfp* [Kan^R^, Cam^R^]	This work
SBR04	MG1655 *ΔrecN::kan* [Kan^R^]	This work; P1 JW5416 × MG1655
SBR07	MG1655 *ΔdusB::kan* [Kan^R^]	This work; P1 JW3228 × MG1655
SBR08	MG1655 *Δhfq::kan* [Kan^R^]	This work; P1 JW4130 × MG1655
SBR09	MG1655 *ΔydeE::kan* [Kan^R^]	This work; P1 JW1527 × MG1655
SBR19	BL21 *ΔrecN::kan* [Kan^R^]	This work; P1 JW5416 × BL21
SBR20	AB1157 *ΔrecN::kan* [Kan^R^]	This work; P1 JW5416 × AB1157
SBR21	CCUG17620 *ΔrecN::kan* [Kan^R^]	This work; P1 JW5416 × CCUG17620
SBR22	BL21 *Δhfq::kan* [Kan^R^]	This work; P1 JW4130 × BL21
SBR23	AB1157 *Δhfq::kan* [Kan^R^]	This work; P1 JW4130 × AB1157
SBR24	CCUG17620 *Δhfq::kan* [Kan^R^]	This work; P1 JW4130 × CCUG17620
SBR25	BL21 *ΔdusB::kan* [Kan^R^]	This work; P1 JW3228 × BL21
SBR26	AB1157 *ΔdusB::kan* [Kan^R^]	This work; P1 JW3228 × AB1157
SBR27	CCUG17620 *ΔdusB::kan* [Kan^R^]	This work; P1 JW3228 × CCUG17620
SBR28	BL21 *ΔydeE::kan* [Kan^R^]	This work; P1 JW1527 × BL21
SBR29	AB1157 *ΔydeE::kan* [Kan^R^]	This work; P1 JW1527 × AB1157
SBR30	CCUG17620 *ΔydeE::kan* [Kan^R^]	This work; P1 JW1527 × CCUG17620

P1 transduction is shown as: P1 Donor × Recipient. Relevant antibiotic resistances are shown in brackets: Amp^R^, Ampicillin resistance; Cam^R^, Chloramphenicol resistance; Kan^R^, Kanamycin resistance; Tet^R^, Tetracycline resistance. ^a^The *recA306* notation refers to the *Δ(srl-recA)306* mutation, which only leaves a small portion of the *recA* gene and can be considered a deletion of *recA* [43–45]. ^b^The SMG3 strain is a variant of the MG1655 wild type.

### Fluorescence microscopy for validation of screening candidate strains

Fixed samples of individually cultured strains were imaged with fluorescence microscopy to assess DNA compaction phenotypes following CIP exposure, ensuring controlled conditions for validation of candidate strains. This approach utilized a Leica DM6000 B microscope equipped with a 100× oil objective (Leica HCX Plan Apochromat 100× 1.40 NA, PH3 CS), a Leica EL6000 metal-halide light source, and a Hamamatsu C9100-14 EM-CCD camera. After treatment and fixation, cells were stained with 5 µg/ml Hoechst 33 258 in PBS for 10 min, washed once with PBS, and concentrated 2–4 times in a PBS resuspension. The stained cells (10 µl) were evenly distributed onto an agar pad pre-made within a Gene Frame (Thermo Scientific no. AB0576) on a glass slide and sealed with a coverslip after drying. Images were captured from 4–6 distinct locations across the agar pad to achieve comprehensive cell representation. Each image included a phase-contrast channel for cell outlines and a fluorescence channel for Hoechst 33 258, with excitation at 334–406 nm and emission collection at 407–497 nm.

### Strain construction for investigation of DNA supercompaction

Strains constructed from screening hit strains to investigate the role of their mutations and deletions in DNA supercompaction are listed in Table 1. Standard P1 transduction procedures [38] were employed to transfer genetic mutations and deletions from these hit strains into various background strains, as well as to introduce *hupA100*::mCherry [39] chromosomally for DNA visualization during live-cell imaging. Electroporation transformation, as detailed in our previous study [18], was used to introduce plasmids required for studying RecN localization and dynamics, and for the SOS response assay. The pSG101 (pSOS) plasmid encoded GFP-RecN regulated by the native RecN promoter [17], while the p*lexA*-*gfp* reporter plasmid encoded GFP regulated by the *lexA* promoter [40, 41]. In cases where both donor and recipient strains carried kanamycin resistance, the donor *kan*-cassette was removed using FLP recombinase (pCP20) [42].

### Live-cell spinning disk microscopy

To explore DNA supercompaction progression and RecN dynamics in selected hit strains, live-cell spinning disk microscopy was employed with the same methodology outlined in [18]. In brief, the setup involved a Nikon Eclipse Ti2-E inverted microscope configured with a 60× oil objective, a CrestOptics X-Light V2 spinning disk confocal module (50:400 µm spinning disk), and a Lumencor Celeste multiline laser, supplemented with two Teledyne Photometrics Kinetix sCMOS cameras. Temperature control was maintained through a stage-top incubator chamber and an objective heater. Strains were individually cultured to exponential phase, then exposed to CIP (10 µg/ml) and incubated for an additional minute. Immediately after, they were transferred onto LB½-agar pads (LB diluted 1:1 with Milli-Q water, with 1% agarose) on microscope slides within a Gene Frame (Thermo Scientific, no. AB0576), sealed with a cover slip (#1.5 thickness). Imaging commenced 10 min after CIP exposure, acquiring images from three locations while maintaining samples at 37°C. For the HU-mCherry-only DNA compaction experiments, images were acquired at 1-min intervals, while 2-min intervals were used for the experiments with GFP-RecN. Fluorescence imaging used a TL channel for cell outlines and an mCherry fluorescence channel for imaging mCherry excited at 546 nm with emission collection between 580 and 610 nm; a GFP fluorescence channel was added for strains with GFP-RecN, with excitation at 477 nm and emission collected between 501 and 521 nm.

### Image analyses to characterize DNA distributions and RecN dynamics

Image processing and analyses followed protocols we established in our previous study [18]. We utilized the open-source software Fiji (ImageJ) [54], together with the plugin MicrobeJ (beta-version 5.13p (20)) [55], to assess DNA supercompaction and RecN dynamics. In brief, images were preprocessed to remove background noise, followed by cell segmentation in MicrobeJ using the “Medial Axis” method, with limitations applied to cell area, length, width, curvature, and sinuosity. DNA profiles were determined by measuring fluorescence intensity along the long axes of segmented cells, either for individual cells or after averaging across a range of cells from the same sample and time point. DNA profile widths were identified as the distance between the outer bounds of the fluorescence peaks at 80% of their maximum intensity. The midcell-to-quarter-position intensity ratio was calculated from averaged DNA profiles as the log2-transformed ratio between fluorescence intensity at midcell (50% of cell length) and the mean fluorescence intensity at the quarter positions (25% and 75% of cell length). These calculations were made and plotted with GraphPad Prism (version 10.4.1) to visualize DNA compaction. Furthermore, MicrobeJ facilitated the creation of kymograph heatmaps and the quantification of RecN foci and their colocalization with HU-mCherry foci. Kymograph heatmaps, generated using the ShapePlot tool, summarized the average fluorescence distribution within cells. GFP-RecN foci were detected within segmented cells for quantification, with their positions compared to HU-mCherry foci locations to assess RecN colocalization with DNA. The resulting data were transferred to GraphPad Prism (version 10.4.1) for plotting.

### SOS response assay

To assess SOS response activity in screening hit strains, the SOS response assay involved transformation of these strains with a p*lexA*-*gfp* reporter plasmid, which conferred either chloramphenicol or kanamycin resistance depending on the strains’ existing resistance genes. The plasmid conveys GFP fluorescence, regulated by the *lexA* promoter, was quantified using flow cytometry. Strains were cultured to exponential growth in LB medium. Samples of 6 µl were collected every 30 min, from just before until 120 min after CIP exposure, when the wild-type control sample’s SOS response activity plateaued. These samples were diluted in 200 µl PBS within wells of a 96-well plate, adjusting the sampled cell culture volume if necessary to achieve ∼2000 events per µl. The plate with diluted samples was immediately brought to an Accuri C6 flow cytometer (BD Bioscience) for measurement of integrated GFP fluorescence signal from 20 000 cells per sample. GFP was excited at 488 nm and emission was collected at 515–545 nm. A detection threshold for forward scatter height was applied to exclude smaller particles from analyses. SOS response activity was quantified by normalizing the sample’s mean GFP fluorescence to the mean forward scatter area (mean cell size), under the assumption that larger cells contain more GFP.

### Reverse transcription-quantitative polymerase chain reaction experiments

Reverse transcription-quantitative polymerase chain reaction (RT-qPCR) was used to quantify *recN* expression in hit strains relative to wild type. The *ΔrecN* strain was also included as a negative control. Strains were cultured individually in LB medium to an OD_600_ around 0.4–0.6 and exposed to CIP. The wild-type sample was split in two just before exposure to maintain an unchallenged control. After 20 min CIP exposure, 1.5 ml of the culture was pelleted at 4°C and resuspended in TE buffer (10 mM Tris, 0.1 mM EDTA, pH 7.5) with 1 mg/ml lysozyme and 2 mg/ml proteinase K while kept on ice. After vortexing, the resuspension was incubated at 25°C for 5 min with shaking for lysis and protein degradation. Total RNA was then isolated using the RNeasy Plus Mini Kit with columns for genomic DNA elimination (Qiagen no. 74 134), according to the manufacturer’s protocol while keeping the sample on ice or 4°C. RNA concentration and integrity was assessed with DNA ScreenTape analysis (Agilent no. 5067–5576 and 5067–5577) using a 4150 TapeStation system (Agilent). RNA samples with concentration above 25 ng/µl and RIN^e^ (RNA integrity number equivalent) above 8.0 were considered acceptable for RT-qPCR analysis. Samples with lower RIN^e^ measures were evaluated for consistent amplification behavior with other biological replicates and only excluded if they deviated notably.

Reverse transcription (RT) was performed using the High-Capacity complementary DNA (cDNA) Reverse Transcription Kit (Applied Biosystems no. 4 368 814) with random primers according to the manufacturer’s instructions. For each 20-µl reaction volume, 250 ng of total RNA was used. Synthesis of cDNA was done with the following program: 25°C for 10 min, 37°C for 120 min, and 85°C for 5 min.

Quantitative PCR (qPCR) was performed on a QuantStudio 5 Real-Time PCR system (Applied Biosystems) with a 384-well setup, using a PowerUp SYBR Green master mix (Applied Biosystems no. A25742). Each 20-µl qPCR reaction contained 0.5 µl of synthesized cDNA (equivalent to 6.25 ng input RNA), and 200 nM of gene-specific primers for *gyrA* (reference gene) or *recN* (Table 2). Two candidate reference genes were evaluated, *gyrA* and *ihfB*, and *gyrA* was selected based on superior expression stability under CIP exposure. All primers were designed using the Primer-BLAST tool [56] to ensure specificity, and checked for secondary structures or self-complementarity with Primer3Plus [57]. They were synthesized by Eurofins Genomics Europe. The qPCR amplification program was: 50°C for 2 min, 95°C for 2 min, 40 cycles of 95°C for 15 s, and 60°C for 1 min; followed by 95°C for 15 s, 60°C for 1 min, and 95°C for 15 s with a temperature increase of 0.15°C/s for the melt curve. No-template controls (NTCs) for both primer pairs and No-RT controls (RNA template without RT) for each biological replicate were included to assess reagent contamination and genomic DNA contamination [58]. All samples and controls were run in the same 384-well plate for qPCR. Melt curves were analyzed to confirm the specificity of each primer pair in all samples. To ensure negligible contamination, we required that NTCs showed no notable amplification or only sporadic late amplification (Cq above 37), and that No-RT controls amplified at least 10 cycles later than the corresponding cDNA samples. Quantification cycle (Cq) values were exported for further analyses.

**Table 2.** List of primers used for RT-qPCR experiment to quantify *recN* and *gyrA* expression

Primer name	Sequence (5′–3′)
recN_p1_1422_Fwd	CAAACTGCTGCGTCAACTCG
recN_p1_1545_Rev	TTCTGTCATCGCACCATCGG
gyrA_p1_1887_Fwd	ACGTATCACTGCGATCCTGC
gyrA_p1_1996_Rev	GACGGTTGAACTCGGTGAGG

Cq values were averaged from three technical replicates within each biological replicate (*n* = 3), and adjusted by subtracting one cycle to account for first-cycle double-strand synthesis from cDNA [58]. Efficiency-corrected expression quantities (Q) were calculated for each gene using efficiencies (E) determined from standard curves (*recN*: slope = −3.221, y-intercept = 19.267, R^2^ = 1, E = 2.044; *gyrA*: slope = −3.205, y-intercept = 19.085, R^2^ = 0.999, E = 2.051) according to $Q = {{E}^{ - ( {Cq - 1} )}}$. *recN* quantities were normalized to *gyrA* within each sample. Each sample’s expression was standardized to the mean of the treated wild type, and the log_2_-transformation of this relative expression (fold change values) was used for statistical analysis.

### UV exposure of hit strains

Hit strains were irradiated with UV to assess DNA supercompaction and strain-dependent survival effects. Each strain’s ONC was diluted to achieve new cultures with an OD_600_ of 0.01 in fresh LB medium containing appropriate antibiotics. These cultures were incubated at 37°C until reaching exponential growth at an OD_600_ around 0.2. Cultures were then split into two subcultures: one remained unchallenged, while the other was UV irradiated. Both subcultures were pelleted by centrifugation at 20 000 × *g* for 30 s at 37°C and resuspended in PBS to prevent UV absorption by the LB medium. Suspensions were gently pipetted onto the center of 90 mm-diameter Petri dishes. The exposed subculture received a UV dose of 5 J/m^2^ at 254 nm using a UV crosslinker (UVP CL-1000). Following UV exposure, subcultures were collected, pelleted by centrifugation, and resuspended in LB medium with appropriate antibiotics. For survival assays, 100 µl of each resuspension underwent serial 10-fold dilution in LB medium within 96-well plates. For imaging, remaining cell suspensions were incubated in a water bath at 37°C with shaking for 15 min before fixation with an equal volume of ethanol. These samples were subsequently imaged using the same fluorescence microscopy approach as employed to validate screening candidate strains.

### Survival assays

Survival assays examined the tolerance of hit strain samples to CIP or UV exposure, relative to unchallenged samples. Strains were cultured until their OD_600_ reached 0.2, at which point cultures were split into two subcultures: one remained unchallenged, while the other was exposed to either CIP or UV. For CIP exposure, a 10 µg/ml dose was employed to assay the time-dependent survival of strains, as detailed in our previous study [18]. Samples were collected from each subculture prior to treatment and at time points ranging from 1 to 60 min after exposure. For UV exposure, wild-type (BW25113) and *ΔrecN* (JW5416) strains were assayed in a dose-dependent manner using UV doses ranging from 5 to 70 J/m^2^, applying the exposure method described earlier. The survival of hit strains was only assayed at a single dose of 5 J/m^2^.

Ten-fold serial dilutions of both exposed and unchallenged subcultures were prepared, with each dilution spotted onto LB-agar plates containing relevant antibiotics for selection. Plates were incubated overnight at 37°C. For each strain, colonies were counted at the lowest dilution where distinguishable colonies appeared, and colony forming units (CFU) per ml were calculated for both subcultures. Relative survival after treatment was expressed as the ratio of CFU/ml between exposed and unchallenged subcultures.

### Statistical analysis

The initial screening was performed in three biological replicates, complemented by re-imaging candidate strains and validation experiments to support hit selection. Details regarding testing of machine learning models and calculation of enrichment scores for screening candidates are provided in the Supplementary Material. The number of biological replicates for follow-up experiments of hit strains is indicated in the corresponding figure captions. We generally used ordinary one-way analysis of variance (ANOVA) with Dunnett correction for multiple comparisons to analyze differences in compaction metrics, *recN* expression, SOS response activity, and survival between hit strains and wild type. A Pearson correlation analysis was performed for analyzing the relationship between DNA supercompaction impairment and CIP sensitivity. For the alternative genetic-background experiment, DNA profile widths were analyzed using one-sample, one-tailed *t*-tests against the predefined threshold of 25% of cell length, representing the upper limit of profile width for completed wild-type-like DNA supercompaction. These analyses were conducted using GraphPad Prism (version 10.4.1). Time- and dose-dependent survival data after CIP exposure and UV irradiation, respectively, were analyzed in R (version 4.4.1). Survival values, relative to unchallenged parallels, were log_10_-transformed and fitted by linear mixed-effects models including only main effects, with random intercepts for biological replicates (see Zenodo repository in the ‘Data availability’ section). Estimates with 95% confidence intervals are presented for fixed effects, adjusted with Dunnett correction for multiple comparisons with wild type. Unless otherwise specified, two-tailed *P*-values below 0.05 were considered significant and indicated with asterisks as follows: *P ≤.05; **P ≤.01; ***P ≤.001; ****P ≤.0001. Figures employ color-coding to visually indicate significant differences between tested strains, while asterisks denote the level of statistical significance.

## Results

### Development of a novel machine learning-assisted high-throughput screening workflow for identifying strains with impaired DNA supercompaction

**Figure 1.**
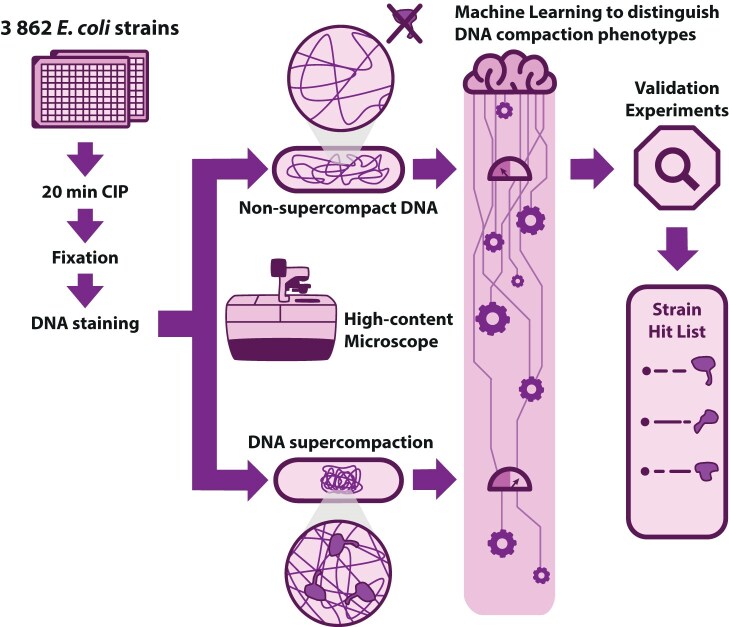
Overview of screening procedure used to identify gene deletions affecting the DNA supercompaction response in *E. coli*. Strains were cultured, exposed to CIP for 20 min (10 µg/ml), fixed, and stained for DNA visualization in batches in 384-well plates before imaging with automated high-content microscopy. A machine learning-assisted CellProfiler pipeline was used to analyze images and classify DNA compaction phenotypes. Strains displaying impaired DNA supercompaction (*ΔrecN*-like or unchallenged compaction phenotypes) were selected as candidates for validation experiments, after which strains with consistently impaired compaction were designated as hits.

**Figure 2.**
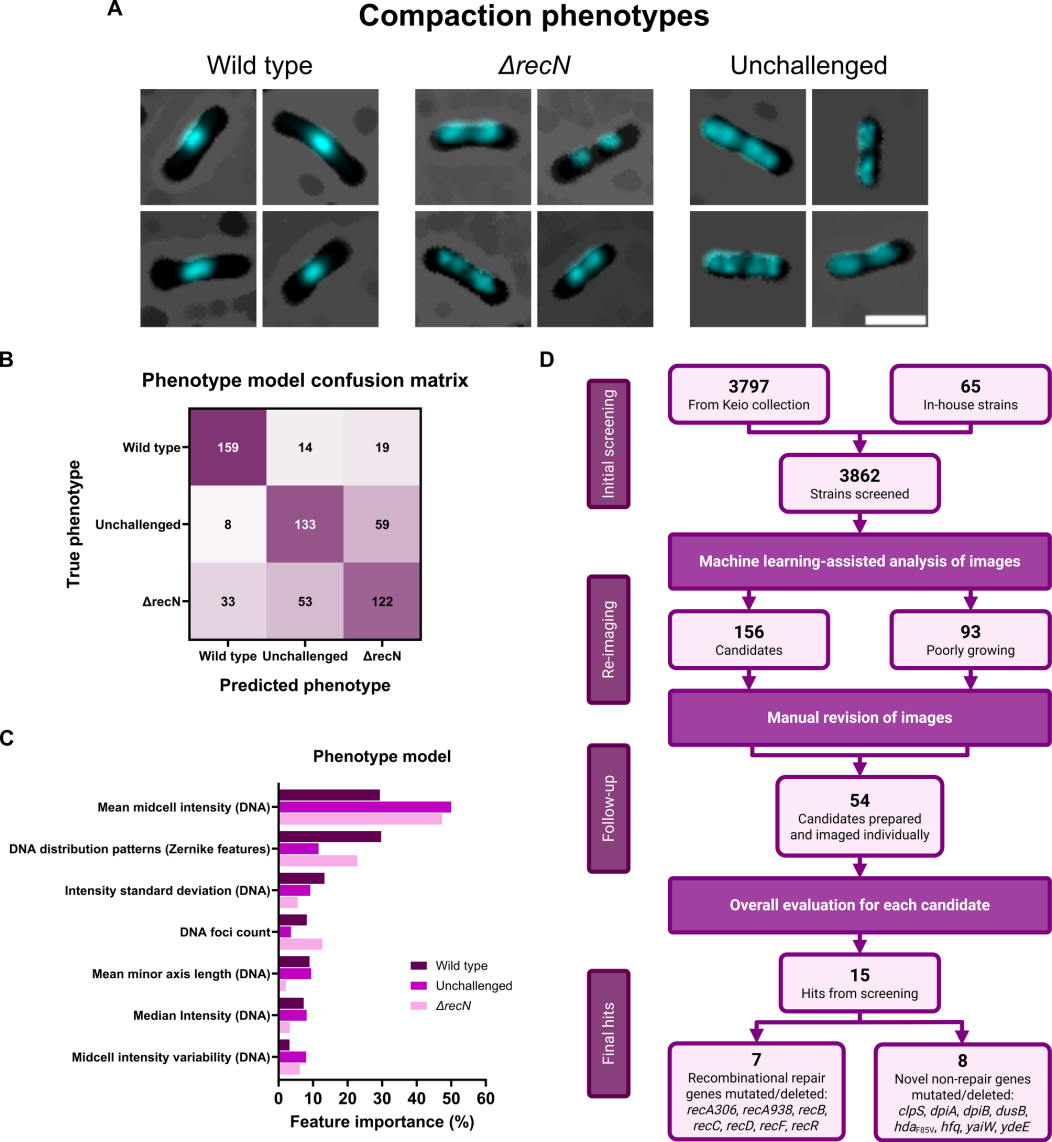
Development of the Phenotype model (**A–C**) and selection process for hit strains from the screening (**D**). (**A**) Representative images of cells from control samples used to train the Phenotype model. Wild-type cells exhibit midcell compaction, the end-point of DNA supercompaction. *ΔrecN* cells display quarter-position compaction, while unchallenged cells show multifocal DNA distribution, both indicating impaired DNA supercompaction when observed in CIP-exposed cells. Scale bar is 3 µm. (**B**) Confusion matrix summarizing the Phenotype model’s prediction performance. The matrix displays the true phenotypes for 200 cells per model-predicted phenotype. (**C**) Feature importance analysis for the Phenotype model showing the relative contribution of different cellular parameters to classification of each compaction phenotype. Parameters are grouped based on similarity to emphasize which cellular features are critical for accurate phenotype differentiation. (**D**) Flowchart summarizing the selection process used to identify hit strains from the screening. Candidate strains identified in the initial screening, along with poorly growing strains, were re-imaged and subjected to follow-up validation experiments, after which strains with consistently impaired DNA supercompaction were designated as hits. The flowchart was created in BioRender; Vikedal, K. (https://BioRender.com/wos5279).

To explore the genetic contributors to DNA supercompaction following CIP exposure, we developed a scalable machine learning-assisted high-throughput screening workflow. Using this workflow, we screened 3797 single-gene deletion *E. coli* strains from the Keio collection [23], along with 65 additional in-house strains (Supplementary Table S1), to select candidate strains with impaired DNA supercompaction for downstream validation (overview of screening procedure in Fig. 1). Our previous work demonstrated that CIP exposure triggers DNA supercompaction in wild-type *E. coli* cells (BW25113), a response in which the nucleoid’s morphology transitions from a multifocal distribution, through quarter-position compaction, to dense midcell compaction [18]. The phenotypic responses of the screened strains were compared to three control sample phenotypes: (i) CIP-exposed wild-type cells, which undergo midcell compaction; (ii) CIP-exposed *ΔrecN* cells, which fail to transition past a form of quarter-position compaction; and (iii) unchallenged wild-type and *ΔrecN* cells, which display multifocal distributions (Fig. 2A).

Strains were cultured to exponential phase in 384-well plates, exposed to 10 µg/ml CIP for 15–20 min, fixed, stained with Hoechst 33 258, and then imaged by high-content microscopy. The imaging produced nearly 50 000 images, which were analyzed in a high-throughput manner using CellProfiler (version 4) [33] with batch processing on a cluster computer (see Supplementary Material). We initially attempted to classify the control sample phenotypes (Fig. 2A) using simple measurements such as DNA foci count and relative area, in line with our previous work [18]. However, under the high-throughput preparation and automated imaging conditions of the screening, reliable segmentation of individual DNA foci proved challenging, and these biologically intuitive measurements were therefore not sufficiently robust for consistent phenotype classification. To overcome this obstacle, we employed machine learning models that used a broader set of image-derived features describing cell morphology and intracellular DNA distribution patterns, which enabled more reliable classification of compaction phenotypes.

We trained two machine learning models on 2000 objects (cells) each from a subset of control sample images: a Single-Cell model to classify segmented objects as either single cells or irrelevant objects, and a Phenotype model to classify the compaction phenotype of the retained single cells (training details in Supplementary Material). Multiple classification strategies were evaluated for each model, and for both models we selected a 15-parameter FastGentleBoosting strategy in CellProfiler Analyst (version 3) [34] because it combined strong performance with reproducible and transparent feature selection, facilitating straightforward interpretation of the measurements used for classification.

Following image preprocessing and cell segmentation, the Single-Cell model was applied to classify segmented objects such as clusters, imaging artefacts, and out-of-focus cells as irrelevant, and exclude them from the subsequent phenotype classification. The Single-Cell model achieved an F_1_ score of 0.84, where F_1_ score is the harmonic mean of precision and recall (see Supplementary Material), and used cellular elongation as its most important feature for classification (Supplementary Fig. S1 and Supplementary Table S2).

The Phenotype model was developed to classify retained single cells according to the control phenotype they most closely matched—wild-type, *ΔrecN*, or unchallenged. These single-cell classifications were then combined at the sample level to determine whether samples were enriched for the wild-type phenotype or for the two phenotypes consistent with impaired DNA supercompaction (see Supplementary Material for details). The model classified the wild-type phenotype well (F_1_ = 0.81), although differentiation between *ΔrecN* and unchallenged phenotypes was less accurate (Fig. 2B; detailed testing results in Supplementary Material). When scoring compaction phenotypes, mean midcell Hoechst 33 258 intensity was the most important feature (Fig. 2C and Supplementary Table S3; feature importance calculation is detailed in Supplementary Material, see also Zenodo repository in the ‘Data availability’ section). The model also incorporated multiple Zernike features [59, 60], which are quantifications of DNA distribution patterns. Together, these sets of features represented 64% of feature importance (Fig. 2C) and captured biologically meaningful differences in intracellular DNA distribution between compaction phenotypes more robustly than simpler metrics alone. While DNA foci count contributed to classification accuracy, it represented only 8% of feature importance (Fig. 2C), indicating that broader DNA distribution features were needed for phenotype classification under the high-throughput conditions of the screening.

To assess the practical performance of the machine learning-assisted classification, we applied the two models to 83 control samples—half of the controls included in the screening dataset. Overall, 63 samples (75.9%) were correctly classified, and among the CIP-exposed wild-type controls, all but one sample were classified correctly. While only two samples (2.4%) were misclassified, 18 (21.7%) remained unclassified. Samples with insufficient phenotype enrichment were intentionally not force-classified, as part of a conservative strategy to reduce the number of false positives. During model development, we also evaluated a two-class phenotype model in which the *ΔrecN* and unchallenged phenotypes were combined into a single impaired-supercompaction class, but this model left substantially more samples unclassified than the three-class Phenotype model used for the screening (details in Supplementary Material).

Because the Phenotype model showed limited separation between the *ΔrecN* and unchallenged phenotypes, and replicate-level reproducibility varied among strains with weaker impaired-supercompaction phenotypes, we used the machine learning-assisted screening as a candidate-selection step within a broader hit-identification workflow. Candidate strains were selected using predefined enrichment thresholds across individual replicates (detailed selection criteria in Supplementary Material). Hit determination then relied on a conservative multistep process that included manual quality control of model predictions for candidate strains, re-imaging of selected strains, and subsequent validation experiments under more controlled growth and preparation conditions (Fig. 2D).

### Conservative validation identifies 15 hit strains with impaired DNA supercompaction

Upon completion of the machine learning-assisted screening and manual quality control of model predictions, 156 candidate strains were selected based on enrichment for impaired-supercompaction phenotypes following CIP exposure (Supplementary Table S4). Additionally, 93 strains displayed poor growth during the screening (Supplementary Table S5; selection criteria in Supplementary Material), which complicated their phenotype classification and necessitated further investigations for accurate compaction phenotype assessment. This observation is consistent with previous reports that some Keio collection strains grow more slowly than the wild type [28, 30, 61]. Together, these strain sets were carried forward through the validation process outlined in Fig. 2D.

The first validation step involved preparation and re-imaging of the 156 candidate strains in a common plate under the high-throughput format. Manual quality control of the images (see Supplementary Material) was used to remove obvious artefacts and samples whose model-predicted impairment was contradicted by clear wild-type-like DNA supercompaction, leaving 35 candidates for further follow-up experiments. For the 93 poorly growing strains, we performed a separate replicate in which all strains were cultured in individual tubes to ensure that they reached exponential growth prior to CIP exposure, fixation, and DNA staining (see Supplementary Material). After high-throughput imaging, 19 additional candidates showed evidence of impaired DNA supercompaction and were selected for follow-up experiments. In total, 54 candidate strains were selected for controlled validation experiments (Supplementary Table S6).

To validate their compaction phenotypes under more controlled conditions, the 54 candidate strains were cultured individually and prepared for imaging on slides. This allowed us to use a fluorescence microscope with an oil immersion objective to improve resolution and better distinguish DNA compaction phenotypes. Imaging was performed on samples grown in both rich LB medium—as in the screening—and in AB minimal medium. The minimal medium reduces the number of replicating chromosomes [37], which we expected would facilitate clearer distinction of compaction phenotypes. After assessing these validation images alongside previously collected data for each candidate strain, those that consistently displayed phenotypes distinct from the wild-type control in the majority of examined cells were identified as the final hit strains.

This conservative screening and validation workflow identified 15 final hit strains with impaired DNA supercompaction (Table 3). These included strains with mutations or deletions of recombinational repair genes, as well as genes not previously linked with DNA compaction or repair, including *ΔclpS, ΔdusB, Δhfq, ΔyaiW, ΔydeE*, and *hda*_F85V_. These six non-DNA-repair hit strains were prioritized for the major follow-up experiments to test whether the screening workflow could identify novel and unexpected contributors to DNA supercompaction. Although the *ΔdpiA* and *ΔdpiB* strains were included among the final hit strains, they were not prioritized for follow-up experiments because their impaired-supercompaction phenotype was restricted to a filamenting subpopulation observed only during growth in rich medium (Supplementary Fig. S2). With these final hits validated, we then investigated the effects of their mutations and deletions on DNA supercompaction and related cellular processes.

**Table 3.** Details on the 15 hit strains with impaired DNA supercompaction from the screening

Strain	Genotype	Details about protein and gene	Source
JW0865	BW25113 ***ΔclpS***	Chaperone binding	Keio collection; [23]
HI1733	SC1148 ***ΔdpiA***	SOS response induction after β-lactam exposure [62], DNA binding	In-house strain; [63]
HI1734	SC1148 ***ΔdpiB***	SOS response induction after β-lactam exposure [62]	In-house strain; [63]
JW3228	BW25113 ***ΔdusB***	tRNA binding and modification, Co-transcribed with fis	Keio collection
KS1115	SMG3^a^ ***hda*_F85V_**	Inhibitor of re-initiation of DNA replication, Possible gain of function mutant [64]	In-house strain
JW4130	BW25113 ***Δhfq***	RNA-binding to stimulate RNA-RNA pairing	Keio collection
STL9253	MG1655 ***recA306*^b^**	SOS response inducer after DNA damage, Homologous recombination protein, DNA binding	In-house strain; [43–45]
ALS972	MG1655 ***recA938***	SOS response inducer after DNA damage, Homologous recombination protein, DNA binding	In-house strain; [53]
JW2788	BW25113 ***ΔrecB***	Homologous recombination protein, DNA binding	Keio collection
JW2790	BW25113 ***ΔrecC***	Homologous recombination protein, DNA binding	Keio collection
JW2787	BW25113 ***ΔrecD***	Homologous recombination protein, DNA binding	Keio collection
JW3677	BW25113 ***ΔrecF***	Homologous recombination protein, DNA binding	Keio collection
JW0461	BW25113 ***ΔrecR***	Homologous recombination protein, DNA binding	Keio collection
JW0369	BW25113 ***ΔyaiW***	Unknown function	Keio collection
JW1527	BW25113 ***ΔydeE***	Dipeptide exporter	Keio collection

^a^The SMG3 strain is a variant of the MG1655 wild type. ^b^The *recA306* notation refers to the *Δ(srl-recA)306* mutation, which only leaves a small portion of the *recA* gene intact and can be considered a deletion of *recA* [43–45].

### Quantitative live-cell analysis of DNA supercompaction impairment in hit strains

**Figure 3.**
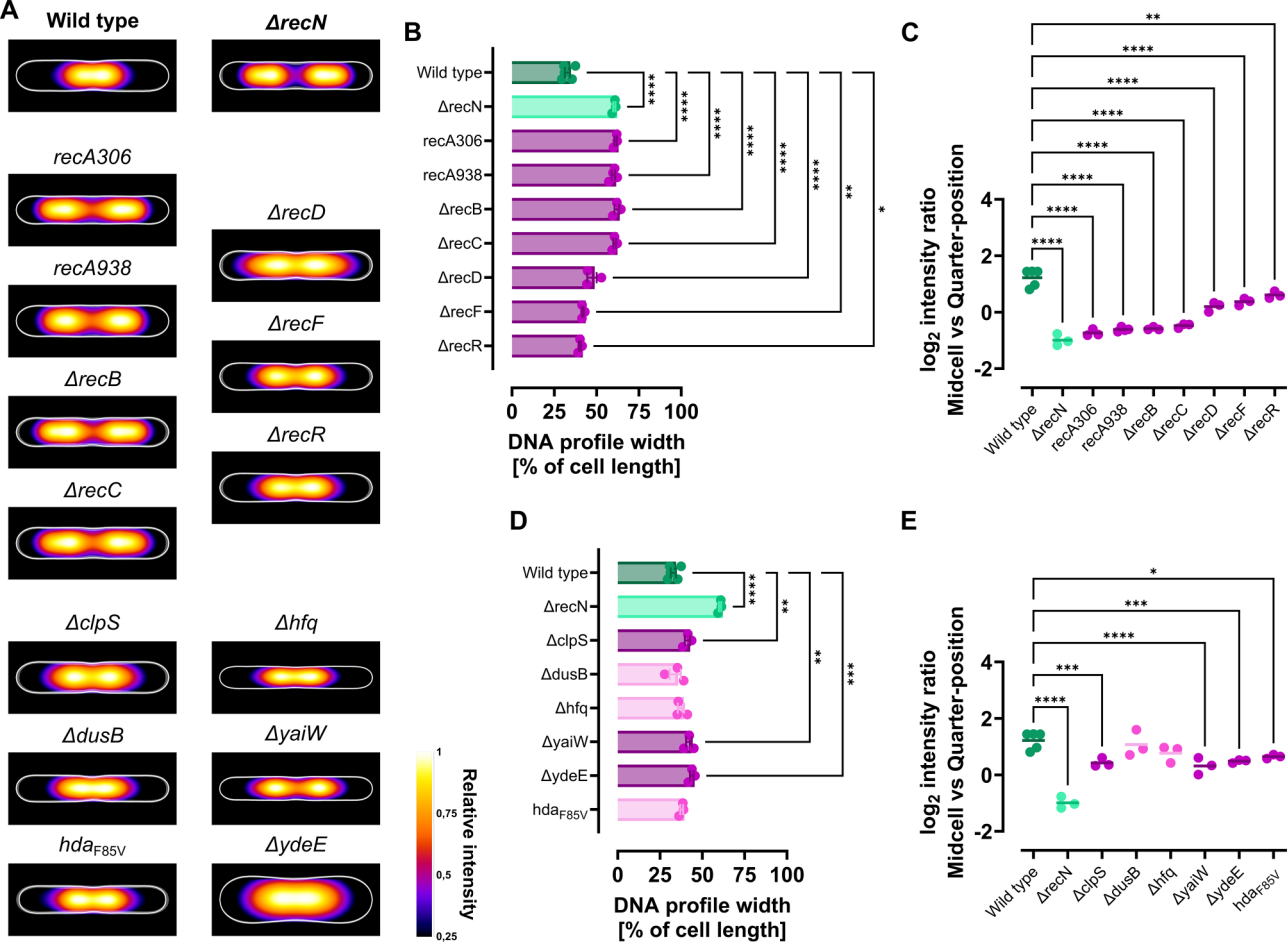
Live-cell analysis of DNA supercompaction in hit strains after 20 min CIP exposure (10 µg/ml). All strains carried endogenous HU-mCherry for DNA visualization and were imaged using live-cell spinning disk microscopy at 1-min intervals; the *recA306* and *recA938* mutations were analyzed after transduction into the HU-mCherry-containing BW25113 background, as listed in Table 1. (**A**) Heatmaps showing relative intensity distributions of HU-mCherry within cells, averaged from 229–1551 cells per strain from representative biological replicates. Wild type and *ΔrecN* are shown for comparison. (**B, D**) DNA profile width measurements for hit strains compared with wild type. Profile widths were quantified relative to the cells’ long axis by measuring the distance between the outer bounds of HU-mCherry fluorescence peaks at 80% of the maximum averaged intensity. (**C, E**) Comparison of DNA compaction using the log_2_-transformed ratio between fluorescence intensities at midcell (50% of cell length) and the mean of the quarter-positions (25% and 75% of cell length). Hit strains with deletions of recombinational repair genes are shown in panels (A)–(C), while hit strains with deletions or mutations of genes not previously associated with DNA compaction or repair are shown in panels (A), (D), and (E). Columns and lines represent means from three to five biological replicates; dots indicate individual replicate values; error bars show standard error of the mean. Wild type is shown in dark green; *ΔrecN* in light green. Strains with values significantly different from wild type are shown in dark magenta; others in light magenta. Ordinary one-way ANOVA with Dunnett correction was used for statistical comparisons; only significant differences are annotated. *P ≤.05; **P ≤.01; ***P ≤.001; ****P ≤.0001.

In the first phase of our follow-up investigation, we used live-cell imaging on hit strains carrying endogenous HU-mCherry for DNA visualization [18, 52] to examine how their mutations and deletions affected DNA supercompaction after CIP exposure. The screening results showed that our original quantification of supercompaction impairment based on DNA foci counts [18] was not sufficiently robust for screening classification, whereas the multifeature Phenotype model performed better. For comparison of DNA supercompaction among the hit strains, however, we wanted simpler quantitative metrics that could be more easily interpreted biologically. Informed by the most important features of the Phenotype model (Fig. 2C), we therefore defined two complementary DNA compaction metrics based on DNA intensity profiles measured along the cells’ long axis: (i) DNA profile width and (ii) the midcell-to-quarter-position intensity ratio. These metrics clearly distinguished the wild-type and *ΔrecN* control strains (Fig. 3), consistent with our previous work establishing RecN as essential for completion of DNA supercompaction [18].

The intracellular DNA distributions of the hit strains differed from wild type to varying degrees after 20 min CIP exposure, as illustrated by the fluorescence-intensity heatmaps in Fig. 3A. These heatmaps are averages of hundreds of cells from representative biological replicates for each strain, collected at a time point when most wild-type cells are expected to have reached midcell compaction [18]. Because cell lengths are normalized in the heatmap panel, apparent differences in cell width reflect variation in cell length. The *ΔydeE* cells were significantly shorter than wild type, whereas *ΔdusB, Δhfq*, and *ΔyaiW* cells were significantly longer at this time point (Supplementary Fig. S3), which should be considered when interpreting the DNA profile widths.

Analysis of compaction metrics calculated from averaged DNA profiles showed that the *recA306, recA938, ΔrecB*, and *ΔrecC* strains had the most severe impairments of DNA supercompaction, and that they resembled the *ΔrecN* control strain in both DNA profile width (Fig. 3B) and midcell-to-quarter-position intensity ratio (Fig. 3C). The *ΔrecD, ΔrecF*, and *ΔrecR* strains also differed significantly from wild type in both metrics, but their impairments were milder than those of the most severely affected strains (Fig. 3B and C). Among the non-DNA-repair hit strains, the differences from wild type were also generally less pronounced. Still, *ΔclpS, ΔyaiW*, and *ΔydeE* strains differed significantly from wild type by both metrics, while the *hda*_F85V_ strain differed significantly by the intensity ratio metric only (Fig. 3D and E). By contrast, *ΔdusB* and *Δhfq* strains were less clearly separated from wild type by these quantitative metrics, although the heatmaps still indicated somewhat altered DNA organization relative to wild type (Fig. 3A), likely a reflection of their increased cell lengths (Supplementary Fig. S3).

To evaluate whether the averaged-DNA-profile analysis from Fig. 3 accurately reflected differences in DNA compaction among hit strains, we also analyzed DNA profile widths at the single-cell level (Supplementary Fig. S4). These analyses supported the same overall interpretation of compaction impairments among the hit strains as the averaged-DNA-profile measurements. Both analytical approaches further indicated that complete DNA supercompaction (midcell compaction) is associated with a reduction in DNA profile width to roughly 15%–25% of the cell length (Supplementary Fig. S5A and B). Additionally, time-resolved analysis of DNA profile widths showed that the most severely impaired strains (*recA306, recA938, ΔrecB, ΔrecC*, and *ΔrecN*) displayed only a subtle decrease in DNA profile width during the first 30 min after CIP exposure, whereas the other hit strains exhibited more marked width reductions during this period (Supplementary Fig. S5C and D).

### Non-DNA-repair hit deletions alter GFP-RecN colocalization with the nucleoid

Next, we asked whether the impaired DNA supercompaction observed in the non-DNA-repair hit strains was associated with altered RecN dynamics, motivated by our previous finding that loss of functional RecA disrupts RecN activity and nucleoid localization after CIP exposure [18]. For this investigation, we introduced the pSOS plasmid into the HU-mCherry-carrying strains used for live-cell compaction analysis to enable GFP-RecN expression at native RecN levels during live-cell imaging [17, 18].

**Figure 4.**
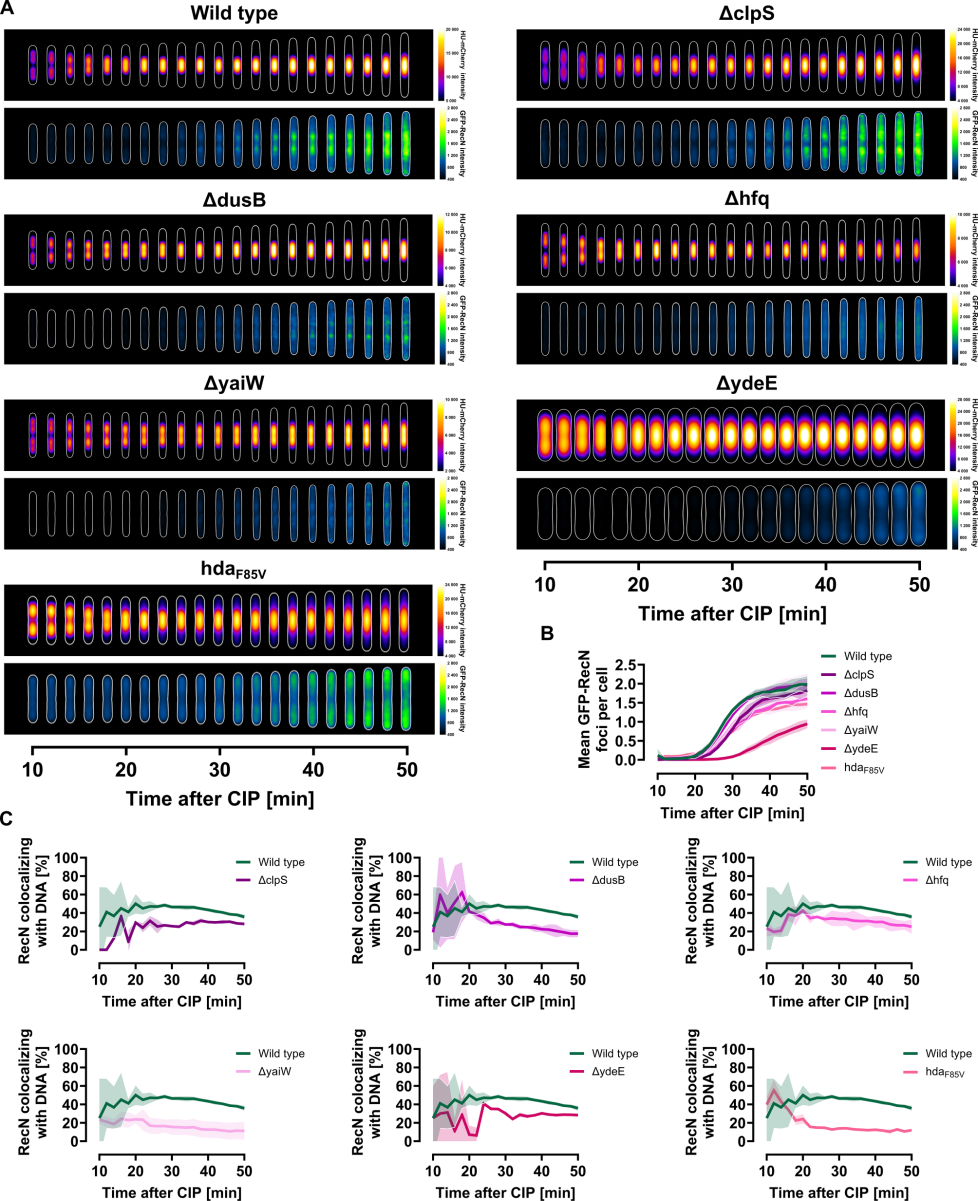
Live-cell analysis of compaction and GFP-RecN dynamics after CIP exposure in wild type and non-DNA-repair hit strains. All strains carried the pSOS plasmid for GFP-RecN expression and were imaged using live-cell spinning disk microscopy at 2-min intervals, starting 10 min after CIP exposure. Data for the wild-type strain (A, B, and C) were reported in our previous study on DNA supercompaction. (**A**) Kymograph heatmaps showing intensity distributions of HU-mCherry (upper panels) and GFP-RecN (lower panels) within cells following CIP exposure. Cell lengths are normalized to the longest cell of each strain; apparent differences in width therefore reflect cell length variation. Note the variations in HU-mCherry intensity scales. Results are from representative biological replicates averaging 105–1807 cells per strain. (**B**) Mean number of GFP-RecN foci per cell over time. (**C**) Percentage of GFP-RecN foci colocalizing with DNA over time for each hit strain compared with wild type. Lines represent means from two biological replicates (four for wild type), with shaded regions indicating standard deviation. Early time points in panel (C) reflect few cells with weak foci, resulting in large standard deviations.

While GFP-RecN foci cluster near the compact nucleoid in wild type cells [18], kymograph heatmaps of intracellular GFP-RecN indicated varying distribution patterns in the non-DNA-repair hit strains, with generally reduced GFP-RecN fluorescence (Fig. 4A). Nevertheless, the number of GFP-RecN foci per cell remained broadly similar over time with wild type for most of these hit strains (Fig. 4B). By analyzing DNA compaction metrics from these images, we found that particularly the *ΔclpS* strain experienced reduced supercompaction impairment with GFP-RecN expression and that it was no longer significantly different from wild type (Supplementary Fig. S6 versus Fig. 3).

Despite the broadly similar GFP-RecN foci numbers, the proportion of these foci colocalizing with the nucleoid was clearly reduced in the non-DNA-repair hit strains relative to wild type (Fig. 4C). Especially the *ΔyaiW* and *hda*_F85V_ strains displayed strong reductions of GFP-RecN nucleoid colocalization. These results indicate that several of the non-DNA-repair hit deletions alter RecN dynamics during CIP-induced DNA supercompaction.

### RecN expression and SOS response activity vary across hit strains

**Figure 5.**
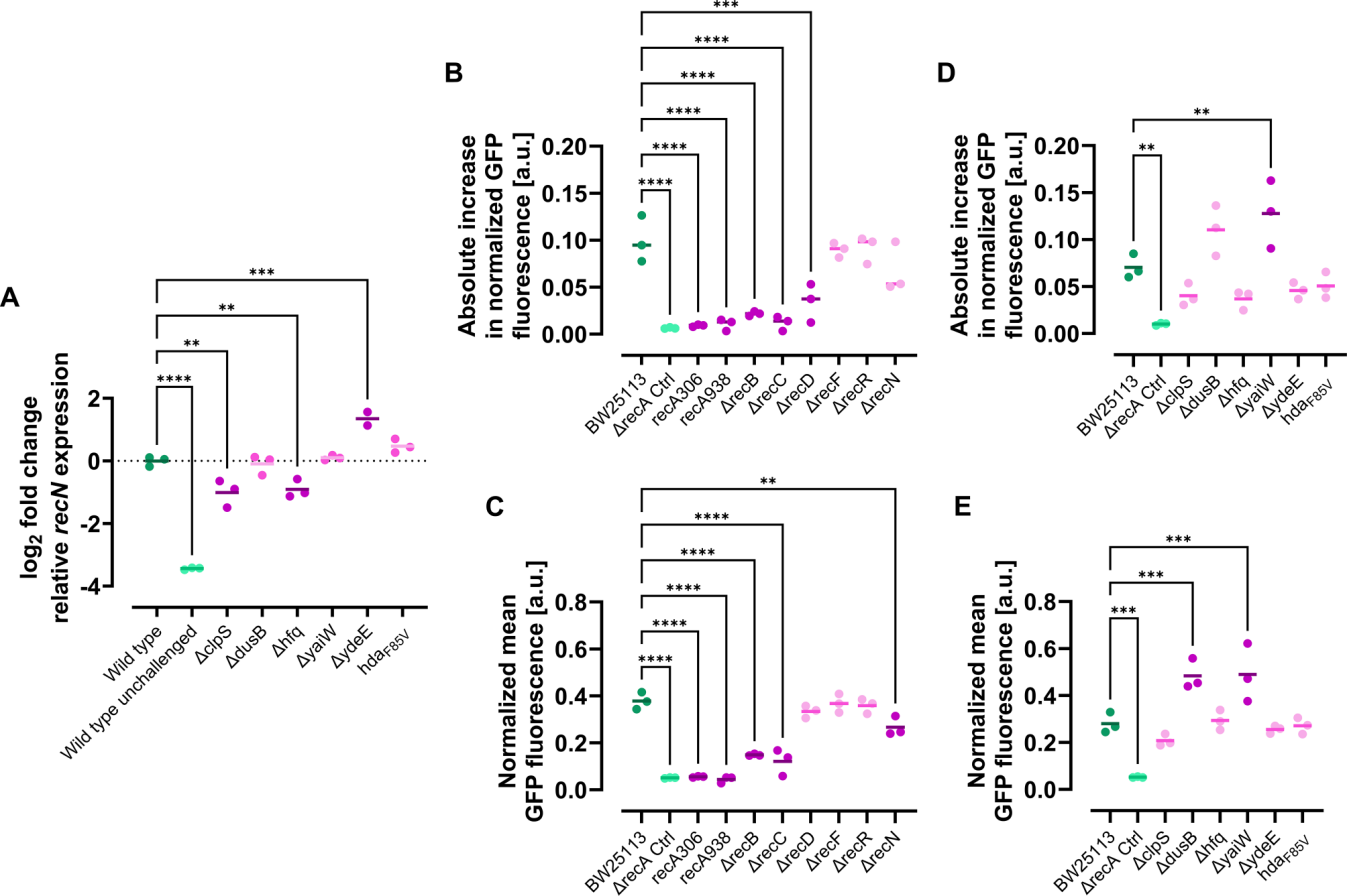
Quantification of *recN* expression (**A**) and SOS response activity (**B–E**) in hit strains after exposure to 10 µg/ml CIP. (**A**) RT-qPCR analysis of *recN* expression in non-DNA-repair hit strains after 20 min CIP exposure. Expression is shown as log_2_ fold change relative to treated wild type after normalization to the reference gene *gyrA*, with an unchallenged wild type as a negative control. All cDNA samples had Cq values between 18.2 and 19.5 for the *gyrA* primer pair, and between 18.9 and 22.8 for the *recN* primer pair. Lines represent means from 2–3 biological replicates; dots indicate individual replicates. (**B–E**) SOS response assay of strains carrying a plasmid with a *lexA* promoter-regulated GFP reporter. Mean GFP fluorescence was quantified by flow cytometry (20 000 cells per sample) and normalized to mean forward scatter area (cell size) as a measurement for SOS response activity. (**B, D**) Absolute change in SOS activity from baseline (0 min) to 30 min after CIP exposure. (**C, E**) SOS activity measured 120 min after exposure. The assay was performed separately for strains with mutations or deletions of recombinational repair genes (B, C), and genes not previously associated with DNA compaction or repair (D, E). A *ΔrecA* strain from the Keio collection (JW2669] served as the negative control. Lines represent means from three biological replicates; dots indicate individual replicate means. Wild type (BW25113) is shown in dark green, negative control in light green. Strains with values significantly different from the corresponding wild type are shown in dark magenta; others in light magenta. Ordinary one-way ANOVA with Dunnett correction was used for statistical comparisons; only significant differences are annotated. **P ≤.01; ***P ≤.001; ****P ≤.0001.

CIP-induced DSBs trigger the SOS response [6], which regulates *recN* expression, particularly in the early part of the response when DNA supercompaction occurs [8, 9, 18]. We therefore investigated next whether the impaired supercompaction phenotypes of the hit strains were associated with altered *recN* expression or SOS response activity (Fig. 5). RT-qPCR was used to quantify *recN* expression in the non-DNA-repair hit strains relative to wild type after 20 min CIP exposure, following normalization to the reference gene *gyrA*. To quantify SOS response activity, we introduced the p*lexA*-*gfp* reporter plasmid into the followed-up hit strains, enabling *lexA* promoter-regulated GFP expression measurable by flow cytometry [40, 41]. GFP fluorescence was sampled just before CIP exposure and at 30-min intervals until 120 min post-exposure, when fluorescence signals plateaued in wild type and other strains (Supplementary Fig. S7A and B). SOS activity was then quantified after normalization to the mean forward scatter, to account for filamentation and presumably higher GFP levels in larger cells.

RT-qPCR analysis showed that *recN* expression varied substantially among the non-DNA-repair hit strains (Fig. 5A) but did not simply mirror the GFP-RecN phenotypes observed in Fig. 4. Whereas the *ΔyaiW* and *hda*_F85V_ strains showed the strongest reductions in GFP-RecN nucleoid colocalization, their *recN* expression did not differ significantly from wild type. Instead, the *ΔclpS* and *Δhfq* strains exhibited significantly lower *recN* expression, while the *ΔydeE* strain had significantly higher *recN* expression.

The SOS response activity assay likewise revealed varying response patterns among the hit strains. Consistent with impaired DSB processing [6, 65], *recA, recB*, and *recC* deletion strains failed to increase their SOS response activities immediately after CIP-induced DNA damage (Fig. 5B) and maintained very low activity levels both at 30 min (Supplementary Fig. S7C) and 120 min post-exposure (Fig. 5C). Whereas RecA has been shown to contribute to DNA supercompaction beyond its role in SOS induction alone [18], the low SOS activity of *ΔrecB* and *ΔrecC* strains may explain their severe supercompaction impairments. In contrast, the *ΔrecD* strain showed only a minor early increase in SOS response activity (Fig. 5B) yet achieved activity levels similar to wild type at 30 min (Supplementary Fig. S7C) and 120 min (Fig. 5C) post-exposure, consistent with its ability to load RecA despite compromised regulation [65]. The *ΔrecN* strain initiated the SOS response like wild type but showed a small yet significant reduction in activity at 120 min post-exposure (Fig. 5C). Among the non-DNA-repair hit strains, most showed SOS activity resembling wild type, though the *ΔdusB* and particularly the *ΔyaiW* strains displayed pronounced increases in SOS response activity levels during the first 30 min of CIP exposure (Fig. 5D) and achieved consistently higher levels than wild type (Fig. 5E and Supplementary Fig. S7D) indicative of sustained elevation of the DNA damage responses. Together with the RT-qPCR results, these findings indicate that impaired DNA supercompaction cannot simply be explained by lowered *recN* expression or reduced SOS response activity, at least among the non-DNA-repair hit strains.

### Severe DNA supercompaction impairment is associated with high CIP sensitivity

Our research previously demonstrated that a *ΔrecN* strain is extremely sensitive to CIP, with survival dropping to undetectable levels after only 1 min of 10 µg/ml CIP exposure [18]. Given that *ΔrecA* strains are also known for their heightened sensitivity to DSBs [28, 66–68], we first confirmed that the *recA306* and *recA938* strains exhibited poor survival after exposure to 10 µg/ml and 20 ng/ml (minimum inhibitory concentration) CIP (Supplementary Fig. S8; *P*-values 0.0037 and 0.0083, respectively; Supplementary Table S7). We then explored whether altered CIP sensitivity extended to the other hit strains by testing their survival after various durations of exposure to 10 µg/ml CIP relative to unchallenged parallel cultures.

**Figure 6.**
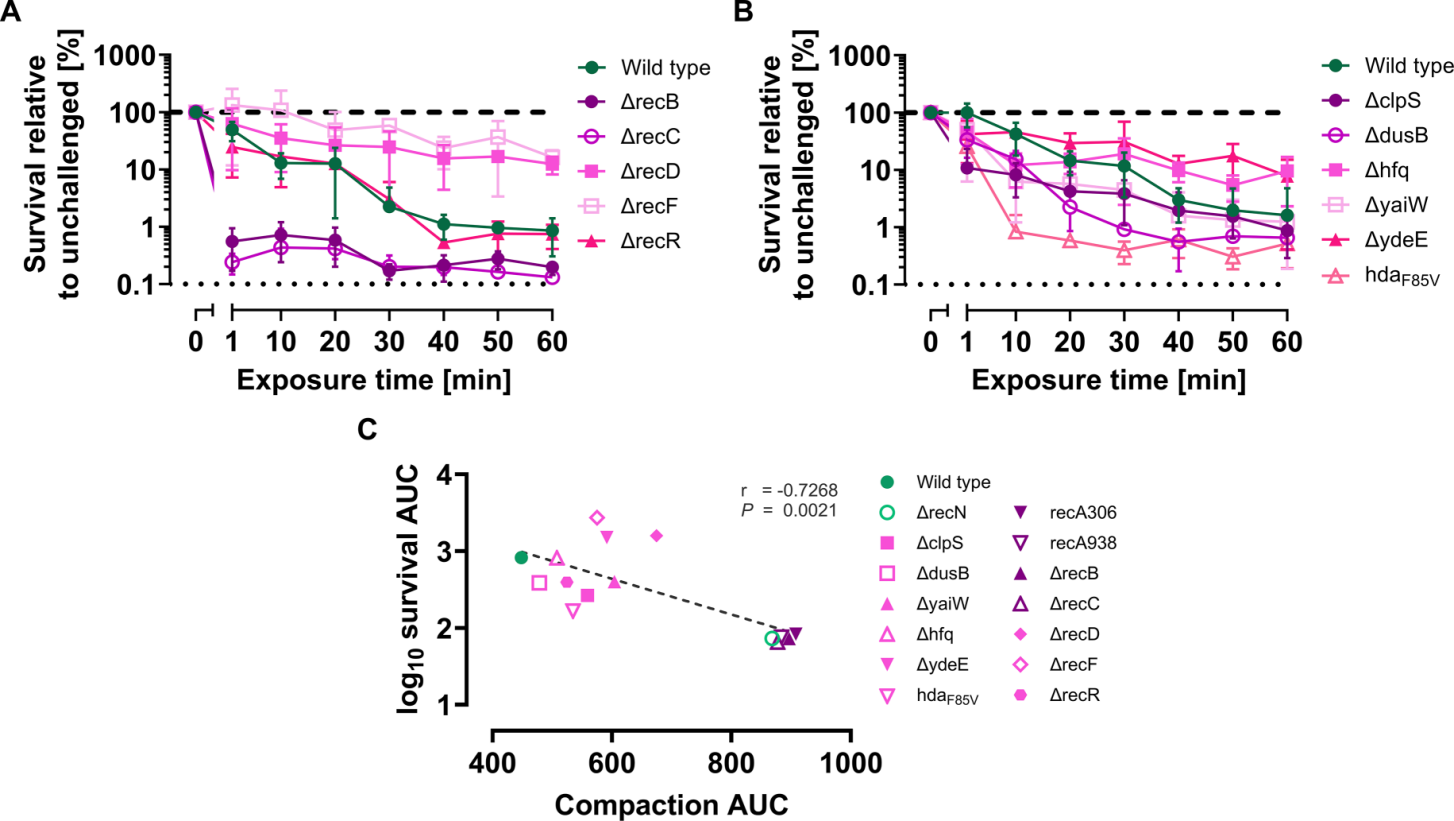
CIP sensitivity varies across hit strains and correlates with impaired DNA supercompaction. (**A, B**) Time-dependent survival after exposure to 10 µg/ml CIP for hit strains with mutations or deletions of recombinational repair genes (**A**) and genes not previously associated with DNA compaction or repair (**B**). Relative survival was calculated from CFU per ml by normalization to an unchallenged parallel culture originating from the same population at baseline (0 min), defining baseline survival as 100% for each strain. Survival was measured from 1–60 min after CIP exposure. Symbols with connecting lines represent means from three to four biological replicates; error bars indicate standard deviations. Thick dashed lines indicate survival of unchallenged parallels; thin dotted lines mark the assay’s detection limit. (**C**) Correlation analysis between impairment of DNA supercompaction and CIP sensitivity across hit strains. DNA supercompaction was quantified as the mean area under the curve (AUC) of DNA profile width measurements at 15–30 min after CIP exposure (see Supplementary Fig. S5), with higher values indicating impaired compaction. CIP sensitivity was quantified as the mean log_10_-transformed AUC of relative survival at 0–60 min after CIP exposure, with lower values indicating increased sensitivity. The dashed line indicates the linear regression fit used for Pearson correlation analysis (r = −0.7268, *P* = 0.0021). Survival data for *ΔrecN, recA306*, and *recA938* are included from Supplementary Fig. S8.

Similar to *ΔrecN* and the *recA* hit strains, *ΔrecB* and *ΔrecC* strains exhibited extreme sensitivity to CIP (Fig. 6A; *P*-values <.0001; Supplementary Table S8), reflecting their inability to initiate DNA repair by processing DSBs [65]. While *ΔrecR* resembled the wild type (*P* = 0.47), *ΔrecD* and *ΔrecF* strains displayed reduced sensitivity compared with wild type (Fig. 6A; *P*-values 0.0080 and <.0001, respectively; Supplementary Table S8). Among the non-DNA-repair hit strains, *hda*_F85V_ exhibited the highest sensitivity, whereas *ΔydeE* was significantly less sensitive than wild type (Fig. 6B; *P* <.0001 and *P* = 0.012, respectively; Supplementary Table S8). The *ΔclpS, ΔdusB*, and *ΔyaiW* strains appeared slightly more sensitive than wild type, while *Δhfq* appeared slightly less sensitive, though none of these differences were statistically significant (Fig. 6B; *P*-values 0.10–0.24; Supplementary Table S8).

To evaluate whether DNA supercompaction impairment was associated with CIP sensitivity across the hit strains, we performed a correlation analysis between AUC metrics quantifying each property (Fig. 6C). This analysis showed a significant correlation between impaired DNA supercompaction and CIP sensitivity (Pearson r = −0.7268, *P* = 0.0021), indicating that severe impairment of DNA supercompaction was associated with higher CIP sensitivity. However, this association was driven mainly by the strains with the most severe compaction impairment—*recA306, recA938, ΔrecB, ΔrecC*, and *ΔrecN*—because they also displayed the most extreme CIP sensitivities (Fig. 6C). Among the remaining hit strains with milder supercompaction impairments, survival phenotypes were more heterogeneous, and the correlation is less clear (Fig. 6C), indicating that supercompaction impairment is not uniformly linked to CIP sensitivity across all followed-up hit strains.

## Discussion

Prompted by our previous discovery that RecN and RecA are essential for DNA supercompaction following severe DNA damage [18], we set out to define the broader genetic landscape of CIP-induced DNA supercompaction. In parallel, we aimed to establish a scalable workflow for identifying bacterial strains with altered DNA organization phenotypes. To achieve these goals, we developed a machine learning-assisted high-throughput screening workflow based on high-content imaging, followed by a conservative validation process (Figs 1 and 2). By applying this workflow to the Keio collection of *E. coli* single-gene deletion strains and additional in-house strains, we identified 15 hit strains with impaired DNA supercompaction (Table 3). These hits featured deletions or mutations affecting known recombinational repair genes, as well as novel genes not previously associated with DNA compaction or repair. The screening provided broad but not exhaustive coverage of the *E. coli* genome, marking an important step toward mapping the genetic contributors to DNA supercompaction.

### Defining DNA supercompaction completion and impairment

Follow-up of the hit strains refined our biological understanding of the DNA supercompaction response. In our previous work, we described DNA supercompaction as an ordered reorganization of the nucleoid from a multifocal distribution, through quarter-position compaction, to dense and persistent midcell compaction [18]. The new compaction metrics presented in the current study provide a more quantitative description of this midcell compaction endpoint and of the phenotypes observed in strains with impaired DNA supercompaction. In wild-type cells, completed midcell compaction was characterized by a narrowing of the DNA profile width to roughly 15%–25% of the cell length, together with a 2–4-fold enrichment of DNA intensity at midcell relative to the quarter positions (Fig. 3, and Supplementary Figs S4 and S5). By contrast, strains with severe supercompaction impairment, such as *ΔrecN*, kept broader DNA distributions, with DNA profile width of ∼40%–60% of the cell length and no enrichment of DNA intensity at midcell (Fig. 3, and Supplementary Figs S4 and S5). These findings establish DNA profile width and the relative midcell-to-quarter-position intensity ratio as simple and practical metrics for distinguishing completed DNA supercompaction from impaired supercompaction phenotypes in future studies.

Using these metrics, the hit strains could be separated into two broad groups. Strains lacking functional RecA, RecB, RecC, or RecN showed severe supercompaction impairments (Fig. 3), and their DNA profile widths changed little during the first 30 min of CIP exposure (Supplementary Fig. S5C). In contrast, the remaining followed-up hit strains generally displayed less pronounced differences from wild type in the compaction metrics (Fig. 3), and all showed notable narrowing of their DNA profile widths after CIP exposure (Supplementary Fig. S5C and D).

### RecN and RecA remain the key orchestrators of DNA supercompaction

Our current investigation has reinforced our understanding of RecN and RecA as the key orchestrators of DNA supercompaction [18], while also clarifying how the initial processing of DNA damage influences this response. Live-cell compaction analysis of the *ΔrecN* control confirmed RecN’s essential role in this process (Fig. 3), while the severe impairments observed for *recA306 and recA938* hit strains are consistent with our previous findings that RecA contributes to DNA supercompaction beyond its role in SOS response induction [18].

While the *ΔrecB* and *ΔrecC* strains also showed severe supercompaction impairment (Fig. 3), it is less clear whether RecB and RecC contribute to DNA supercompaction beyond their established roles in processing CIP-induced DSBs and loading RecA for SOS response initiation and subsequent homologous recombination [6, 65]. Their impairment may be explained by their consistently low SOS response activity (Fig. 5C and Supplementary Fig. S7). In line with this interpretation, the *ΔrecD* strain, which lacks the final component of the RecBCD complex but can maintain DSB processing and RecA loading in a dysregulated manner [65], displayed wild-type-like SOS response activity (Fig. 5C and Supplementary Fig. S7) and less pronounced supercompaction impairment than *ΔrecB* and *ΔrecC* (Fig. 3). Nevertheless, the mildly impaired supercompaction phenotype of the *ΔrecD* strain was still significantly different from wild type, indicating that the way in which DSBs are processed and RecA is loaded may influence the timing or extent of DNA supercompaction.

To examine whether the importance of the RecBCD proteins depended on the presence of DSBs, we also assessed DNA compaction after mild UV irradiation, which primarily generates bulky adducts resulting in DNA single-strand gaps [69, 70]. While CIP-induced DSBs rely on RecBCD processing, UV-induced single-strand gaps require the RecFOR proteins for RecA loading and subsequent SOS response initiation and DNA repair [71]. Consistently, the *ΔrecB* and *ΔrecC* strains showed no detectable compaction impairment relative to wild type after UV irradiation (Supplementary Fig. S9). In contrast, while the *ΔrecR* hit strain displayed mild supercompaction impairment after CIP exposure (Fig. 3), its DNA compaction was clearly impaired after UV irradiation (Supplementary Fig. S9), at a level similar to the *recA306, recA938*, and *ΔrecN* strains. These findings suggest that proper DNA damage processing and RecA loading are important prerequisites for DNA supercompaction, while the specific pathway involved depends on the type of DNA lesion. Thus, RecBCD and RecFOR appear to influence the supercompaction process mainly by enabling RecA-dependent damage responses.

To gain a similar context for interpreting the CIP survival assay, we also investigated survival after UV irradiation (Supplementary Fig. S10). After CIP exposure, supercompaction impairment correlated with increased sensitivity across the hit strains (Fig. 6C), but this association was mainly driven by the *recA306, recA938, ΔrecB, ΔrecC*, and *ΔrecN* strains, all of which are also defective in central aspects of DSB repair [11–15]. Their extreme CIP sensitivity may therefore reflect repair defects that are partly or fully disconnected from their roles in DNA supercompaction. In contrast, there was no apparent correlation between supercompaction impairment and CIP sensitivity among the remaining hit strains (Fig. 6C), and only the *recA306* and *recA938* strains displayed significantly different UV sensitivity from wild type (Supplementary Fig. S10). The *ΔrecN* strain showed only a small, nonsignificant increase in UV sensitivity compared with wild type across a range of doses despite its impaired UV-induced DNA compaction (Supplementary Fig. S10C; *P* = 0.06; Supplementary Table S9). Thus, impaired DNA compaction does not generally predict sensitivity across genotoxic agents.

### Non-DNA-repair hit genes appear to have indirect or modulatory contributions to DNA supercompaction

Several genes identified in our screening have not previously been associated with DNA compaction or repair, prompting us to consider how they might influence DNA supercompaction by integrating our experimental data with established literature. Notably, the non-DNA-repair hit strains displayed milder supercompaction impairments than strains lacking functional RecA, RecB, RecC, or RecN (Fig. 3). Several of these non-DNA-repair defects affected RecN dynamics (Fig. 4), *recN* expression (Fig. 5A), SOS response activity (Fig. 5B–E), or survival (Fig. 6) following CIP exposure. However, these effects were heterogeneous and did not reveal a single explanatory pattern for impaired DNA supercompaction. Together, the milder impairments and diverse follow-up results indicate that these genes influence the supercompaction response indirectly or in modulatory roles, rather than acting as core contributors to the DNA reorganization process.

The heterogeneity among the non-DNA-repair hit strains was particularly evident when comparing RecN-associated phenotypes with SOS response activity. All tested non-DNA-repair hit strains showed reduced proportions of GFP-RecN foci colocalizing with the nucleoid after CIP exposure, despite most of them displaying GFP-RecN foci numbers comparable to wild type (Fig. 4). The largest reductions in GFP-RecN nucleoid colocalization were observed for *ΔdusB, ΔyaiW*, and *hda*_F85V_, although none of these strains expressed *recN* at levels significantly different from wild type (Figs 4 and 5A). Moreover, *ΔdusB* and *ΔyaiW* showed elevated SOS response activity, whereas *hda*_F85V_ retained wild-type-like SOS activity (Fig. 5B–E). Despite these partially overlapping RecN-associated and SOS activity phenotypes, the corresponding compaction phenotypes differed: *ΔyaiW* showed clear supercompaction impairment, whereas *ΔdusB* and *hda*_F85V_ were less distinctly separated from wild type in the compaction metrics (Fig. 3). The remaining strains *ΔclpS, Δhfq*, and *ΔydeE* further illustrated this lack of a simple phenotypic pattern, as they showed distinct combinations of altered *recN* expression, GFP-RecN dynamics, and compaction phenotypes (Figs 3–5). Together, these results suggest that SOS activity or *recN* expression alone is not necessarily predictive of DNA supercompaction impairment after CIP exposure, at least when strains retain detectable SOS activity and *recN* expression. Similarly, some non-DNA-repair hit strains displayed increased CIP sensitivity and other showed reduced sensitivity, without any clear relationship between supercompaction impairment and CIP sensitivity among these strains (Fig. 6).

The known or predicted functions of the non-DNA-repair hit genes suggest several possible indirect or modulatory links to DNA supercompaction. The *yaiW* and *ydeE* genes are predicted to encode outer [72] and inner [73] membrane-associated proteins, respectively. Both *yaiW* and *ydeE* are regulated by CpxR [73, 74], while *yaiW* is also part of the σ^E^ regulon [75]. Since CpxR and σ^E^ are key regulators of membrane stress responses, these hits suggest a possible connection between DNA supercompaction and membrane-associated stress physiology. This possibility is further supported by the inner membrane localization of Hfq [76] and our previous observation that deletion of the inner membrane peptide DinQ impairs DNA compaction after UV irradiation [77]. Hfq may also influence the supercompaction response through additional routes, including effects on CIP uptake [78–80] and post-transcriptional regulation of RecB levels [81], although our data do not distinguish between these possibilities. Additionally, the tRNA-modifying role of DusB [82, 83] points to a possible effect on DNA supercompaction through translational modulation. Together, these connections support the view that the non-DNA-repair hits may influence DNA supercompaction through indirect or modulatory effects that alter the physiological state in which RecN- and RecA-dependent DNA reorganization occurs.

As phenotypic effects of gene deletions can depend on genetic background through epistatic and compensatory effects [84–86], the relatively mild and heterogeneous supercompaction impairments observed for the non-DNA-repair hit strains urged us to examine selected deletions in alternative *E. coli* wild-type backgrounds. We transduced *dusB, hfq, recN*, and *ydeE* deletions into the wild-type strains MG1655, AB1157, BL21, and CCUG17620 and assessed their effects on CIP-induced DNA supercompaction (Supplementary Fig. S11). While *recN* deletion consistently impaired supercompaction across all backgrounds in accordance with RecN’s essential role in this process, the effects of *dusB, hfq*, and *ydeE* deletions varied more strongly between genetic environments. This variation supports the interpretation that these non-DNA-repair genes influence DNA supercompaction in a context-dependent manner, consistent with indirect or modulatory roles.

Notably, *dusB* and *hfq* deletions caused more substantial supercompaction impairments in the clinical reference background CCUG17620 [50] than in the more lab-adapted backgrounds [46–49, 51] (Supplementary Fig. S11). This observation led us to examine whether DNA supercompaction also occurs in clinical isolates of *E. coli, Klebsiella oxytoca*, and *Klebsiella pneumoniae*. All tested isolates displayed clear midcell compaction after CIP exposure (Supplementary Fig. S12), indicating that DNA supercompaction is also relevant for the clinical setting as a bacterial response to severe DNA damage. Continued exploration of this response and its underlying molecular mechanisms will be needed to determine whether it influences DNA damage tolerance, antibiotic sensitivity, or resistance development in clinically relevant contexts.

### Novel machine learning-assisted high-throughput screening workflow enables scalable analysis of imageable DNA organization phenotypes

To explore the genetic landscape of DNA supercompaction, we first needed a screening workflow that could assess DNA organization phenotypes at genome scale. High-content imaging enabled us to capture nearly 50 000 images across the Keio collection and additional in-house strains, but this scale also made manual evaluation or low-throughput analytical approaches impractical. Moreover, the DNA foci count-based classification approach used in our previous work [18] was not sufficiently robust under these conditions, as high-throughput preparation, fixation, and automated imaging made reliable segmentation of DNA foci challenging. Accordingly, we developed machine learning models that used a broader set of image-derived features describing cell morphology and intracellular DNA distribution patterns to more reliably classify compaction phenotypes (Fig. 2). Because the Phenotype model did not perfectly distinguish all impaired-supercompaction phenotypes and replicate-level reproducibility varied for strains with mild impairments, the screening was used as an initial candidate-selection step. The full screening workflow developed for this study therefore incorporated a conservative validation process, including manual quality control, re-imaging of candidates, and subsequent validation experiments under more controlled conditions, to identify the final hit strains with impaired DNA supercompaction (Figs 1 and 2D).

The Phenotype model from our screening workflow also helped refine how DNA supercompaction could be quantified in follow-up experiments. Although DNA foci count was biologically intuitive, it accounted for only 8% of feature importance for phenotype classification in the model (Fig. 2C). Instead, features related to midcell DNA intensity and broader DNA distribution patterns (Zernike features) accounted for the majority of feature importance when distinguishing wild-type-like midcell compaction from impaired phenotypes (Fig. 2C). This observation indicated that robust quantification of DNA supercompaction should focus less on counting segmented DNA foci and more on measuring how DNA is distributed along the cell. Thus, we defined two complementary DNA compaction metrics: DNA profile width and the midcell-to-quarter-position intensity ratio, both calculated from DNA intensity profiles measured along the cells’ long axes. These simple quantitative metrics translated the cellular features emphasized by the Phenotype model into biologically interpretable measurements suitable for statistical comparison of DNA supercompaction impairment.

Despite these strengths, the screening workflow had limitations that should be considered when interpreting the list of hit strains. First, the Keio collection enabled broad genome-scale coverage of single-gene deletions, but it does not allow systematic assessment of essential genes, since these cannot be deleted due to lethality [23]. Second, single-gene deletion effects can be masked or confounded by background-specific epistatic or compensatory effects, secondary mutations, growth differences, or functional redundancy [28, 30, 61, 84, 87]. Third, the efficient high-throughput preparation and imaging conditions in the screening introduced some variation in focus quality and signal-to-noise ratio, which could complicate nucleoid lobe distinction and subsequent phenotype classification. Fourth, the machine learning models depended on manually annotated training data. Although we strived to create diverse and representative datasets, annotation errors or biases in these data could propagate to the output classifications [35, 88–90]. Finally, the Phenotype model itself showed limited separation between the *ΔrecN*-like and unchallenged phenotypes (Fig. 2B). Together, these limitations meant the screening workflow was not ideal for distinguishing strains with subtle supercompaction impairments or comprehensively assessing the contributions of all genes in this response. As an example, the *ΔrecA* strain from the Keio collection was notably absent from the candidate list, though subsequent investigations showed that *recA* deletion strains, including the Keio collection strain, consistently failed to compact their nucleoids after 20 min of CIP exposure (Supplementary Fig. S13). Moreover, the candidate list from the initial screening included false-positive and ambiguous candidates. The workflow’s conservative validation process ensured the identification of strains with reproducibly impaired DNA supercompaction.

Though the screening workflow may not have uncovered all genetic contributors to DNA supercompaction, it nevertheless enabled us to identify multiple strains with reproducibly altered DNA organization phenotypes. Future studies using complementary approaches will be needed to identify additional contributors to this response. Importantly, the workflow was not limited to identifying hit strains but also helped reveal which image-derived features were most informative for distinguishing compaction phenotypes and guided the development of quantitative metrics for follow-up analyses. More broadly, adapted versions of this scalable workflow could be used to investigate other imageable DNA organization phenotypes, either for identifying strains with altered phenotypes or for defining which image features best distinguish predefined phenotypic states.

## Supplementary Material

gkag573_Supplemental_Files

## Data Availability

All images from high-content imaging and the analysis results from the screening are available in the BioImage Archive [91] under accession number S-BIAD2152 at https://doi.org/10.6019/S-BIAD2152. Other data underlying this article will be shared on reasonable request to the corresponding author. All pipelines, scripts, and reference documents used for the machine learning-assisted image analysis in this study are available in the Zenodo repository at https://doi.org/10.5281/zenodo.15866742. This Zenodo repository also contains scripts for analyzing classification parameter importance, and scripts and data used for statistical analyses in R.

## Notes

Present address: Nextera AS, 0349 Oslo, Norway
