# Supplementary material for: Exploring the genetic landscape of ciprofloxacin-induced DNA supercompaction in Escherichia coli
Source: Nucleic Acids Res. 2026 Jun 22;54(12):gkag573. doi: 10.1093/nar/gkag573 (PMC13284718; doi:10.1093/nar/gkag573)
Supplement: gkag573_Supplemental_Files [file gkag573_supplemental_files.zip › RevSupplementaryData_KV_ScreeningPaper_Submission.pdf]

SUPPLEMENTARY DATA

Exploring the genetic landscape of ciprofloxacin-induced DNA supercompaction in *Escherichia coli*

Krister Vikedal[1,2], Natalia Berges[1,†], Ida Mathilde Marstein Riisnæs[1,2], Synnøve Brandt Ræder[1], Jørgen Vildershøj Bjørnholt[1,2], Magnar Bjørås[1,2,3], Kirsten Skarstad[1], Emily Helgesen[1,3] and James Alexander Booth[1,3,*]

[1] Department of Microbiology, Oslo University Hospital, Rikshospitalet, 0373 Oslo, Norway
[2] Department of Microbiology, University of Oslo, 0316 Oslo, Norway
[3] Department of Clinical and Molecular Medicine, Norwegian University of Science and Technology, 7030 Trondheim, Norway
† Current affiliation: Nextera AS, 0349 Oslo, Norway
* To whom correspondence should be addressed. Email: James.booth@ntnu.no

Table S1. All 65 in-house strains included in the screening. Relevant antibiotic resistances are shown in brackets: Cam^R, Chloramphenicol resistance; Kan^R, Kanamycin resistance; Tet^R, Tetracycline resistance. [a] The SMG3 and ALO1208 strains are variants of the MG1655 wildtype. [b] The *recA306* notation refers to the *Δ(srl-recA)306* mutation, which only leaves a small portion of the *recA* gene and can be considered a deletion of *recA* (1–3).

Strain	Genotype	Source
JABXII-72	BW25113 ***ΔagrB*::*Kan*** [Kan^R]	In-house strain; deletion from (4)
SF169	UF301 ***asnB3057*::*Tet*** [Tet^R]	In-house strain; (5, 6)
JAB003-F8	BW25113 ***ΔatpBE*::*Kan*** [Kan^R]	In-house strain
CAG12077 / CGSC#7347	MG1655 ***crcA* (*pagP*)::*Tn10*** [Tet^R]	(7, 8)
MYU001	MG1655 ***crfC*::*Kan*** [Kan^R]	(9)
GM2927	AB1157 ***dam-13*::*Tn9*** [Cam^R]	(10)
GM3819	AB1157 ***dam-16*** [Kan^R]	(11)
YYH607	YYH605 ***ΔdatA*::*Kan*** [Kan^R]	In-house strain; (12)
NL40	DS941 ***Δdif*** [Kan^R]	(13)
JAB003-B8	BW25113 ***ΔdinQ*::*Kan*** [Kan^R]	In-house strain; deletion from (4)
BM751	CM735 ***dnaA204 clpX1*::*Kan*** [Kan^R]	(14)
BM750	CM735 ***dnaA204 lon*::*Tet*** [Tet^R]	(14)
SMG379	MG1655 ***dnaA***$_{A345S}$***::Tn10*** [Tet^R]	(15)

Strain	Genotype	Source
KS1117	SMG3[a] ***dnaA$_{N346D}$*** [Cam^R]	In-house strain; mutation from (16)
SS1512	MG1655 ***dnaC809 zji202::Tn10*** [Tet^R]	In-house strain; (17)
JAB003-G8	BW25113 ***dnaN$_{G157C}$::Cam*** [Cam^R]	In-house strain; mutation from (15)
JAB004-A1	BW25113 ***dnaN$_{G157C}$::Cam agrB::Kan*** [Cam^R, Kan^R]	In-house strain; mutations from (4, 15)
SMG380	MG1655 ***dnaN$_{G157C}$::Cam*** [Cam^R]	(15)
JAB003-G3	BW25113 ***dnaN$_{H175Y}$::Tet*** [Tet^R]	In-house strain
KS1114	SMG3[a] ***dnaN$_{Q156S}$*** [Cam^R]	In-house strain; mutation from (16)
HI1733	SC1148 ***dpiA::Kan*** [Kan^R]	(18)
HI1734	SC1148 ***dpiB::Kan*** [Kan^R]	(18)
WM2016	CSH26 ***fis::Kan*** [Kan^R]	(19)
LZ1608	C600 ***gyrA$_{S83L}$*** [Tet^R]	(20)
LZ3099	C600 ***gyrA$_{S83L, D87Y}$*** [Tet^R]	(21, 22)
KS1115	SMG3[a] ***hda$_{F85V}$*** [Tet^R]	In-house strain; mutation from (16)
ALO1387	ALO1208[a] ***himD (ihfB)::cat*** [Cam^R]	In-house strain; (23)
ALO1415	ALO1208[a] ***hupA16::Kan*** [Kan^R]	In-house strain; (23)
JAB003-E9	BW25113 ***ΔibsA::Kan*** [Kan^R]	In-house strain
JAB003-E10	BW25113 ***ΔibsB::Kan*** [Kan^R]	In-house strain
JAB003-E11	BW25113 ***ΔibsC::Kan*** [Kan^R]	In-house strain
JAB003-E12	BW25113 ***ΔibsD::Kan*** [Kan^R]	In-house strain
JAB003-F1	BW25113 ***ΔibsE::Kan*** [Kan^R]	In-house strain
JAB003-B7	BW25113 ***ΔistR::Kan*** [Kan^R]	In-house strain
JAB003-F4	BW25113 ***ΔldrD::Kan*** [Kan^R]	In-house strain
CAG18475	MG1655 ***metC162::Tn10*** [Tet^R]	(7)
CM735*ΔmukB*	CM735 ***ΔmukB::Kan*** [Kan^R]	(24)
FR680	MG1655 ***mutD5 (dnaQ) zae13::Tn10*** [Tet^R]	(25)
GM7552	GM7330 ***ΔmutH461::cat*** [Cam^R]	(26)
ME125	MG1655 ***mutH::Kan mutL218::Tn10*** pBAD24-***MutH$_{E56A}$***	(27)

Strain	Genotype	Source
	P*lac*-*yfp*-***mutL-cat*** [Kan^R, Tet^R, Cam^R]	
KM52	AB1157 ***mutL460*::*Cam*** [Cam^R]	(28)
ES1301	***mutS201*::*Tn5*** [Kan^R]	(29)
STL7742	MG1655 ***obgE*::*Tn5*** [Kan^R]	(2)
JAB003-F3	BW25113 ***ΔohsC*::*Kan*** (#1) [Kan^R]	In-house strain
JABXII-74	BW25113 ***ΔohsC*::*Kan*** (#2) [Kan^R]	In-house strain
AQ7677	MC1061 ***priA1*::*Kan*** [Kan^R]	(30)
TWx8	MG1655 ***pyrB*::*Tn5*** [Kan^R]	In-house strain; (31)
JAB003-F5	BW25113 ***ΔrdlD*::*Kan*** [Kan^R]	In-house strain
STL9253	MG1655 ***recA306^b*::*Tn10*** [Tet^R]	In-house strain; (1–3)
ALS972	MG1655 ***recA938*::*Cam*** [Cam^R]	(32)
SMP6-346	MG1655 ***recF*::*Tn5*** [Kan^R]	In-house strain
SS1211	MG1655 ***Δrep*::*Cam*** [Cam^R]	(5)
IF61	MOR166 ***ΔsdiA762*::*Kan*** [Kan^R]	In-house strain; mutation from (33, 34)
YYH609	YYH605 ***ΔseqA*::*Tet*** [Tet^R]	In-house strain; (12)
SS211	MG1655 pBIP-Kan ***seqAΔ10*** [Kan^R]	(35)
UF340	AB1157 ***seqA2 asnB3057*::*Tn10*** [Tet^R]	(6, 36)
SF122	CM735 ***seqA4 asnB3057*::*Tn10*** [Tet^R]	In-house strain; (6)
JAB003-F2	BW25113 ***ΔshoB*::*Kan*** (#1) [Kan^R]	In-house strain
JABXII-73	BW25113 ***ΔshoB*::*Kan*** (#2) [Kan^R]	In-house strain
JAB003-F7	BW25113 ***ΔspoT*::*Kan*** [Kan^R]	In-house strain; deletion from (34)
JAB003-B6	BW25113 ***ΔtisB*::*Kan*** [Kan^R]	In-house strain
LBB925	LBB451 ***tolC*::*Tn10*** [Tet^R]	In-house strain
MG1655-C122	MG1655 ***umuC122*::*Tn5*** [Kan^R]	In-house strain; (37)
DS984	DS941 ***xerC*::*Mu*** [Cam^R]	(13, 38)
DS9008	DS941 ***xerD*::*Tn10*** [Tet^R]	(13, 39)

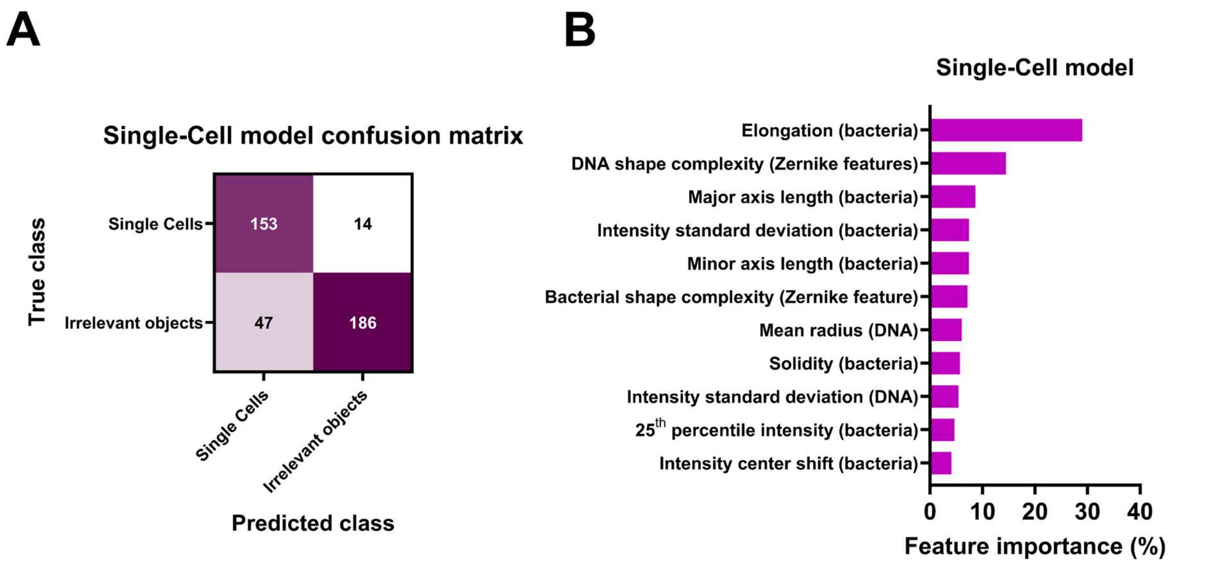

Figure S1. The Single-Cell model was trained to distinguish single cells from irrelevant objects to remove noise from subsequent compaction phenotype analysis. (**A**) Confusion matrix summarizing the Single-Cell model's prediction performance. The matrix displays the true class for 200 cells per model-predicted class. (**B**) Feature importance analysis for the Single-Cell model showing the relative contribution of different cellular parameters to the distinction of single cells from irrelevant objects. Parameters are grouped based on similarity to emphasize which cellular features are critical for accurate distinction of single cells.

Table S2. Parameters used in the Single-Cell model, defining criteria for classifying single cells versus irrelevant objects. Each parameter's associated CellProfiler module and measurement are described, along with interpretations of the assessed cellular features. All measurements were performed on segmented objects (cells). Classification weights are provided for single cell (S) and irrelevant object (I) classes, based on measurements above (true) and below (false) threshold values. Thresholds are rounded to four decimals; weights to two decimals.

Rule #	CellProfiler module and measurement	Feature interpretation	Threshold	Weight (true)	Weight (false)
1	AreaShape-Eccentricity	Elongation (bacteria)	0.8676	S: +0.50<br>I: -0.50	S: -0.86<br>I: +0.86
2	RadialDistribution-ZernikeMagnitude-EnhancedDNAImage-2-0	DNA shape complexity (Zernike features)	0.0022	S: +0.26<br>I: -0.26	S: -0.93<br>I: +0.93
3	Mean-DNAObjects-AreaShape-MeanRadius	Mean radius (DNA)	1.7241	S: +0.17<br>I: -0.17	S: -0.56<br>I: +0.56
4	AreaShape-MinorAxisLength	Minor axis length (bacteria)	14.0563	S: -0.71<br>I: +0.71	S: +0.19<br>I: -0.19
5	AreaShape-MajorAxisLength	Major axis length (bacteria)	17.6847	S: +0.09<br>I: -0.09	S: -0.95<br>I: +0.95
6	AreaShape-Eccentricity	Elongation (bacteria)	0.9306	S: +0.29<br>I: -0.29	S: -0.27<br>I: +0.27
7	AreaShape-Solidity	Solidity (bacteria)	0.8298	S: +0.33<br>I: -0.33	S: -0.36<br>I: +0.36
8	Intensity-StdIntensity-RawBacterialImage	Intensity standard deviation (bacteria)	0.0004	S: +0.09<br>I: -0.09	S: -0.81<br>I: +0.81
9	Mean-DNAObjects-AreaShape-Zernike-2-0	DNA shape complexity (Zernike features)	0.1033	S: +0.15<br>I: -0.15	S: -0.40<br>I: +0.40
10	AreaShape-Eccentricity	Elongation (bacteria)	0.8103	S: +0.07<br>I: -0.07	S: -0.90<br>I: +0.90
11	AreaShape-Zernike-3-1	Bacterial shape complexity (Zernike feature)	0.0045	S: -0.09<br>I: +0.09	S: +0.77<br>I: -0.77
12	Intensity-MassDisplacement-RawBacterialImage	Intensity center shift (bacteria)	0.2507	S: -0.35<br>I: +0.35	S: +0.14<br>I: -0.14
13	AreaShape-Eccentricity	Elongation (bacteria)	0.9555	S: +0.46<br>I: -0.46	S: -0.14<br>I: +0.14

Rule #	CellProfiler module and measurement	Feature interpretation	Threshold	Weight (true)	Weight (false)
14	Intensity-LowerQuartileIntensity-EnhancedBacteriaimage	25th percentile intensity (bacteria)	0.0121	S: +0.42<br>I: -0.42	S: -0.14<br>I: +0.14
15	Intensity-StdIntensity-DNAImage	Intensity standard deviation (DNA)	0.0036	S: +0.07<br>I: -0.07	S: -0.58<br>I: +0.58

Table S3. Parameters used in the Phenotype model, defining criteria for classifying the different DNA compaction phenotypes. Each parameter's associated CellProfiler module and measurement are described, along with interpretations of the assessed cellular features. All measurements were performed on single cells identified by the Single-Cell model. Classification weights are provided for the wild-type (W), unchallenged (U) and ΔrecN (R) phenotypes, based on measurements above (true) and below (false) threshold value. Thresholds are rounded to four decimals; weights to two decimals.

Rule #	CellProfiler module and measurement	Feature interpretation	Threshold	Weight (true)	Weight (false)
1	RadialDistribution-MeanFrac-EnhancedDNAImage-1of6	Mean midcell intensity (DNA)	2.9628	W: +0.66<br>U: -0.96<br>R: -0.70	W: -0.79<br>U: -0.05<br>R: -0.16
2	RadialDistribution-MeanFrac-EnhancedDNAImage-1of6	Mean midcell intensity (DNA)	2.0326	W: +0.00<br>U: -0.57<br>R: +0.18	W: -0.46<br>U: +0.49<br>R: -0.48
3	Children-SingleCellDNAObjects-Count	DNA foci count	1	W: -0.43<br>U: +0.05<br>R: +0.25	W: +0.51<br>U: -0.20<br>R: -0.48
4	Intensity-StdIntensity-SingleCellDNAImage	Intensity standard deviation (DNA)	0.0084	W: +0.48<br>U: -0.29<br>R: -0.21	W: -0.43<br>U: +0.13<br>R: +0.08
5	Mean-SingleCellDNAObjects-AreaShape-MinorAxisLength	Mean minor axis length (DNA)	6.0858	W: -0.20<br>U: +0.06<br>R: +0.01	W: +0.83<br>U: -0.59<br>R: -0.11
6	Intensity-MedianIntensity-EnhancedDNAImage	Median intensity (DNA)	0.0104	W: -0.64<br>U: +0.37<br>R: -0.14	W: +0.20<br>U: -0.19<br>R: +0.05
7	RadialDistribution-ZernikeMagnitude-EnhancedDNAImage-8-4	DNA distribution pattern (Zernike features)	0.0003	W: -0.36<br>U: -0.01<br>R: +0.09	W: +0.32<br>U: +0.04<br>R: -0.17
8	RadialDistribution-RadialCV-RawDNAImage-1of6	Midcell intensity variability (DNA)	0.0614	W: +0.13<br>U: -0.30<br>R: +0.18	W: -0.23<br>U: +0.25<br>R: -0.18
9	RadialDistribution-ZernikeMagnitude-SingleCellDNAImage-3-1	DNA distribution pattern (Zernike features)	0.0028	W: +0.36<br>U: +0.02<br>R: -0.17	W: -0.31<br>U: -0.01<br>R: +0.08
10	RadialDistribution-MeanFrac-RawDNAImage-1of6	Mean midcell intensity (DNA)	1.4091	W: -0.08<br>U: -0.07<br>R: +0.10	W: +0.46<br>U: +0.39<br>R: -0.56
11	RadialDistribution-ZernikeMagnitude-EnhancedDNAImage-5-3	DNA distribution pattern (Zernike features)	0.0002	W: -0.12<br>U: +0.01<br>R: +0.05	W: +0.65<br>U: -0.11<br>R: -0.48

Rule #	CellProfiler module and measurement	Feature interpretation	Threshold	Weight (true)	Weight (false)
12	RadialDistribution-MeanFrac-RawDNAImage-1of6	Mean midcell intensity (DNA)	3.1113	W: +0.89<br>U: -1.00<br>R: -0.85	W: -0.04<br>U: +0.02<br>R: +0.03
13	RadialDistribution-ZernikeMagnitude-SingleCellDNAImage-9-5	DNA distribution pattern (Zernike features)	0.0008	W: -0.60<br>U: +0.14<br>R: +0.09	W: +0.14<br>U: -0.04<br>R: -0.03
14	Intensity-StdIntensity-SingleCellDNAImage	Intensity standard deviation (DNA)	0.0064	W: +0.23<br>U: -0.11<br>R: -0.02	W: -0.39<br>U: +0.10<br>R: +0.02
15	Mean-SingleCellDNAObjects-AreaShape-Zernike-2-2	DNA distribution pattern (Zernike features)	0.1138	W: +0.39<br>U: -0.34<br>R: +0.13	W: -0.16<br>U: +0.07<br>R: -0.03

Table S4. The 156 initial candidate strains from the screening. [a] The *recA306* notation refers to the *Δ(srl-recA)306* mutation, which only leaves a small portion of the *recA* gene and can be considered a deletion of *recA* (1–3).

Strain	Genotype	Source
JW3713	BW25113 ***ΔatpH***	Keio collection (34)
JW0865	BW25113 ***ΔclpS***	Keio collection
JW1241	BW25113 ***Δcls* (*clsA*)**	Keio collection
JW1106	BW25113 ***ΔcobB***	Keio collection
JW2406	BW25113 ***ΔcysZ***	Keio collection
GM3819	AB1157 ***dam-16***	In-house strain; (11)
JW3351	BW25113 ***ΔdamX***	Keio collection
JW0613	BW25113 ***ΔdcuC***	Keio collection
JW0090	BW25113 ***ΔddlB***	Keio collection
JW3670	BW25113 ***ΔdgoK***	Keio collection
JW5187	BW25113 ***ΔdhaK***	Keio collection
JW0054	BW25113 ***ΔdjlA***	Keio collection
JW3228	BW25113 ***ΔdusB***	Keio collection
JW2661	BW25113 ***ΔemrB***	Keio collection
JW1077	BW25113 ***ΔfabH***	Keio collection
JW5020	BW25113 ***ΔfadE***	Keio collection
JW4191	BW25113 ***Δfbp***	Keio collection
JW1379	BW25113 ***ΔfeaR***	Keio collection
JW0149	BW25113 ***ΔfhuB***	Keio collection
WM2016	CSH26 ***fis::Kan***	In-house strain; (19)
JW1058	BW25113 ***ΔflgM***	Keio collection
JW1907	BW25113 ***ΔfliA***	Keio collection
JW4112	BW25113 ***ΔfrdD***	Keio collection
JW3869	BW25113 ***ΔfrvX***	Keio collection
JW1413	BW25113 ***ΔgapC***	Keio collection
JW3841	BW25113 ***ΔglnA***	Keio collection
JW3896	BW25113 ***ΔglpX***	Keio collection
JW3530	BW25113 ***ΔglyS***	Keio collection
JW3297	BW25113 ***ΔgspO***	Keio collection
JW0173	BW25113 ***ΔhlpA* (*skp*)**	Keio collection
JW2965	BW25113 ***ΔhybO***	Keio collection

Strain	Genotype	Source
JW2470	BW25113 ***ΔhyfE***	Keio collection
JW4221	BW25113 ***ΔidnR***	Keio collection
JW0076	BW25113 ***ΔilvI***	Keio collection
JW0687	BW25113 ***ΔkdpF***	Keio collection
JW0071	BW25113 ***ΔleuC***	Keio collection
JW0429	BW25113 ***Δlon***	Keio collection
JW1041	BW25113 ***ΔlpxL***	Keio collection
JW1145	BW25113 ***ΔmcrA***	Keio collection
JW5338	BW25113 ***ΔmdtA***	Keio collection
JW0341	BW25113 ***ΔmhpD***	Keio collection
CM735*ΔmukB*	CM735 ***ΔmukB*::*Kan***	In-house strain; (24)
JW4128	BW25113 ***ΔmutL***	Keio collection
JW3002	BW25113 ***ΔnudF***	Keio collection
JW5375	BW25113 ***ΔnuoC***	Keio collection
JW2280	BW25113 ***ΔnuoE***	Keio collection
JW2279	BW25113 ***ΔnuoF***	Keio collection
JW2278	BW25113 ***ΔnuoG***	Keio collection
JW2277	BW25113 ***ΔnuoH***	Keio collection
STL7742	MG1655 ***obgE*::*Tn5***	In-house strain; (2)
JW0912	BW25113 ***ΔompF***	Keio collection
JW1113	BW25113 ***ΔpepT***	Keio collection
JW3985	BW25113 ***Δpgi***	Keio collection
JW1110	BW25113 ***ΔpotC***	Keio collection
JW0104	BW25113 ***ΔppdD***	Keio collection
STL9253	MG1655 ***recA306*[a]::*Tn10***	In-house strain; (1–3)
ALS972	MG1655 ***recA938*::*Cam***	In-house strain; (32)
JW2788	BW25113 ***ΔrecB***	Keio collection
JW2790	BW25113 ***ΔrecC***	Keio collection
JW2787	BW25113 ***ΔrecD***	Keio collection
JW3677	BW25113 ***ΔrecF***	Keio collection
JW5855	BW25113 ***ΔrecQ***	Keio collection
JW0461	BW25113 ***ΔrecR***	Keio collection
JW3946	BW25113 ***ΔrplK***	Keio collection
JW3907	BW25113 ***ΔrpmE***	Keio collection

Strain	Genotype	Source
JW1707	BW25113 ***ΔrpmI***	Keio collection
JW3039	BW25113 ***ΔrpoD***	Keio collection
JW3169	BW25113 ***ΔrpoN***	Keio collection
JW0387	BW25113 ***ΔsbcC***	Keio collection
JW0388	BW25113 ***ΔsbcD***	Keio collection
JW0711	BW25113 ***ΔsdhC***	Keio collection
JAB003-F2	BW25113 ***ΔshoB::Kan*** (#1)	In-house strain
JW3245	BW25113 ***Δsmg***	Keio collection
JW1264	BW25113 ***ΔsohB***	Keio collection
JW5404	BW25113 ***ΔsseB***	Keio collection
JW5273	BW25113 ***ΔsufB***	Keio collection
JW2985	BW25113 ***ΔsufI*** **(*ftsP*)**	Keio collection
JW1425	BW25113 ***ΔtehA***	Keio collection
JW0979	BW25113 ***ΔtorT***	Keio collection
JW4019	BW25113 ***ΔuvrA***	Keio collection
JW5892	BW25113 ***ΔyadB*** **(*gluQ*)**	Keio collection
JW0349	BW25113 ***ΔyaiO***	Keio collection
JW0362	BW25113 ***ΔyaiT***	Keio collection
JW0366	BW25113 ***ΔyaiV*** **(*iprA*)**	Keio collection
JW0369	BW25113 ***ΔyaiW***	Keio collection
JW0370	BW25113 ***ΔyaiY***	Keio collection
JW0492	BW25113 ***ΔybbS*** **(*allS*)**	Keio collection
JW0527	BW25113 ***ΔybcD*** **(*peaD*)**	Keio collection
JW0532	BW25113 ***ΔybcK***	Keio collection
JW0702	BW25113 ***ΔybgK*** **(*pxpC*)**	Keio collection
JW5106	BW25113 ***ΔybiM*** **(*mcbA*)**	Keio collection
JW0910	BW25113 ***ΔycbL*** **(*gloC*)**	Keio collection
JW0924	BW25113 ***ΔycbT*** **(*elfG*)**	Keio collection
JW0942	BW25113 ***ΔyccR (sxy)***	Keio collection
JW5142	BW25113 ***ΔycdR*** **(*pgaB*)**	Keio collection
JW1042	BW25113 ***ΔyceA (trhO)***	Keio collection
JW1074	BW25113 ***ΔyceD***	Keio collection
JW1246	BW25113 ***ΔyciB***	Keio collection
JW5196	BW25113 ***ΔyciO***	Keio collection
JW1313	BW25113 ***ΔycjW***	Keio collection

**Strain**	**Genotype**	**Source**
JW5207	BW25113 ***ΔydaQ* (*xisR*)**	Keio collection
JW5215	BW25113 ***ΔydbJ***	Keio collection
JW5228	BW25113 ***ΔydcM* (*insQ*)**	Keio collection
JW1459	BW25113 ***ΔyddE***	Keio collection
JW1527	BW25113 ***ΔydeE***	Keio collection
JW1530	BW25113 ***ΔydeJ***	Keio collection
JW5243	BW25113 ***ΔydeN***	Keio collection
JW1637	BW25113 ***ΔydhK***	Keio collection
JW1659	BW25113 ***ΔydhT***	Keio collection
JW5271	BW25113 ***ΔydhX***	Keio collection
JW5274	BW25113 ***ΔydiN***	Keio collection
JW5293	BW25113 ***ΔyeaV***	Keio collection
JW5313	BW25113 ***ΔyedO* (*dcyD*)**	Keio collection
JW2088	BW25113 ***ΔyegW***	Keio collection
JW2400	BW25113 ***ΔyfeR***	Keio collection
JW2421	BW25113 ***ΔyfeU* (*murQ*)**	Keio collection
JW5458	BW25113 ***ΔygeK***	Keio collection
JW3040	BW25113 ***ΔygjF* (*mug*)**	Keio collection
JW3057	BW25113 ***ΔygjQ***	Keio collection
JW3092	BW25113 ***ΔyhaC***	Keio collection
JW3125	BW25113 ***ΔyhbS***	Keio collection
JW3129	BW25113 ***ΔyhbW***	Keio collection
JW3255	BW25113 ***ΔyhdN***	Keio collection
JW3454	BW25113 ***ΔyhiI***	Keio collection
JW3557	BW25113 ***ΔyiaU***	Keio collection
JW3631	BW25113 ***ΔyicI***	Keio collection
JW3689	BW25113 ***ΔyidZ***	Keio collection
JW3831	BW25113 ***ΔyihE* (*srkA*)**	Keio collection
JW5568	BW25113 ***ΔyihV***	Keio collection
JW3899	BW25113 ***ΔyiiU* (*zapB*)**	Keio collection
JW3936	BW25113 ***ΔyijD***	Keio collection
JW3971	BW25113 ***ΔyjaA***	Keio collection
JW3989	BW25113 ***ΔyjbH***	Keio collection
JW4017	BW25113 ***ΔyjbQ***	Keio collection
JW4018	BW25113 ***ΔyjbR***	Keio collection

Strain	Genotype	Source
JW4026	BW25113 ***ΔyjcE***	Keio collection
JW4042	BW25113 ***ΔyjcQ*** **(*mdtO*)**	Keio collection
JW4105	BW25113 ***ΔyjeJ***	Keio collection
JW4124	BW25113 ***ΔyjeS*** **(*queG*)**	Keio collection
JW4148	BW25113 ***ΔyjfP***	Keio collection
JW4271	BW25113 ***ΔyjhR***	Keio collection
JW5968	BW25113 ***ΔyjhX*** **(*topAI*)**	Keio collection
JW4354	BW25113 ***ΔyjjK*** **(*ettA*)**	Keio collection
JW4365	BW25113 ***ΔyjjY***	Keio collection
JW5039	BW25113 ***ΔykgI*** **(*rclB*)**	Keio collection
JW5133	BW25113 ***ΔymcD*** **(*gfcA*)**	Keio collection
JW1154	BW25113 ***ΔymgC***	Keio collection
JW5230	BW25113 ***ΔyncN*** **(*hicA*)**	Keio collection
JW5244	BW25113 ***ΔyneL***	Keio collection
JW5251	BW25113 ***ΔynfO***	Keio collection
JW5331	BW25113 ***ΔyoeB***	Keio collection
JW2460	BW25113 ***ΔypfJ***	Keio collection
JW3111	BW25113 ***ΔyraH***	Keio collection
JW3356	BW25113 ***ΔyrfB*** **(*hofO*)**	Keio collection
JW4167	BW25113 ***ΔytfE***	Keio collection
JW4169	BW25113 ***ΔytfG*** **(*qorB*)**	Keio collection

Table S5. All 93 poorly growing strains from the screening that were re-imaged. [a] The SMG3 and ALO1208 strains are variants of the MG1655 wildtype.

Strain	Genotype	Source
JW3234	BW25113 ***ΔacrF***	Keio collection (34)
JW2201	BW25113 ***Δada***	Keio collection
JW1228	BW25113 ***ΔadhE***	Keio collection
JW4049	BW25113 ***ΔalsB***	Keio collection
JW4047	BW25113 ***ΔalsC***	Keio collection
JW4046	BW25113 ***ΔalsE***	Keio collection
JW4111	BW25113 ***ΔampC***	Keio collection
JW3470	BW25113 ***ΔarsC***	Keio collection
JW5239	BW25113 ***Δbdm***	Keio collection
JW0305	BW25113 ***ΔbetI***	Keio collection
JW2869	BW25113 ***ΔbglA***	Keio collection
CAG12077 / CGSC#7347	MG1655 ***crcA* (*pagP*)::*Tn10***	In-house strain; (7, 8)
JW2639	BW25113 ***ΔcsiR* (*glaR*)**	Keio collection
JW1863	BW25113 ***ΔcutC***	Keio collection
JW2416	BW25113 ***ΔcysW***	Keio collection
JW3905	BW25113 ***ΔcytR***	Keio collection
JW5592	BW25113 ***ΔdapF***	Keio collection
BM750	CM735 ***dnaA204 lon*::*Tet***	In-house strain; (14)
SMG379	MG1655 ***dnaA*** $_{A345S}$**::*Tn10***	In-house strain; (15)
HI1733	SC1148 ***dpiA*::*Kan***	In-house strain; (18)
HI1734	SC1148 ***dpiB*::*Kan***	In-house strain; (18)
JW3509	BW25113 ***ΔdppF***	Keio collection
JW1506	BW25113 ***Δego* (*lsrA*)**	Keio collection
JW2365	BW25113 ***ΔemrK***	Keio collection
JW2434	BW25113 ***ΔeutB***	Keio collection
JW2437	BW25113 ***ΔeutG***	Keio collection
JW2366	BW25113 ***ΔevgA***	Keio collection
JW3065	BW25113 ***ΔexuR***	Keio collection
JW4250	BW25113 ***ΔfecB***	Keio collection
JW1070	BW25113 ***ΔflgL***	Keio collection
JW1328	BW25113 ***Δfnr***	Keio collection

**Strain**	**Genotype**	**Source**
JW3333	BW25113 ***ΔfrlA***	Keio collection
LZ1608	C600 ***gyrA*** $_{S83L}$	In-house strain; (20)
JW1950	BW25113 ***ΔhchA***	Keio collection
KS1115	SMG3[a] ***hda*** $_{F85V}$	In-house strain
JW4130	BW25113 ***Δhfq***	Keio collection
ALO1387	ALO1208[a] ***himD*** **(*ihfB*)::*cat***	In-house strain; (23)
JW0645	BW25113 ***ΔhscC***	Keio collection
JW3903	BW25113 ***ΔhslV***	Keio collection
JW3903	BW25113 ***ΔhybA***	Keio collection
JW3978	BW25113 ***ΔiclR***	Keio collection
JW0895	BW25113 ***ΔihfB***	Keio collection
JW3313	BW25113 ***ΔkefB***	Keio collection
JW0334	BW25113 ***ΔlacY***	Keio collection
JW0720	BW25113 ***ΔmngA***	Keio collection
JW3573	BW25113 ***ΔmtlA***	Keio collection
FR680	MG1655 ***mutD5*** **(*dnaQ*)** ***zae13*::*Tn10***	In-house strain; (25)
JW3193	BW25113 ***ΔnanT***	Keio collection
JW2712	BW25113 ***ΔnlpD***	Keio collection
JW4033	BW25113 ***ΔnrfC***	Keio collection
JW5875	BW25113 ***ΔnuoB***	Keio collection
JW2273	BW25113 ***ΔnuoL***	Keio collection
JW4064	BW25113 ***ΔphnE***	Keio collection
JW1555	BW25113 ***ΔrelE***	Keio collection
JW3753	BW25113 ***ΔrhlB***	Keio collection
JW3756	BW25113 ***Δrho***	Keio collection
JW1072	BW25113 ***ΔrluC***	Keio collection
JW3947	BW25113 ***ΔrplA***	Keio collection
JW4161	BW25113 ***ΔrplI***	Keio collection
JW3261	BW25113 ***ΔrpmJ***	Keio collection
JW3134	BW25113 ***ΔrpsO***	Keio collection
JW2132	BW25113 ***ΔsanA***	Keio collection
JW1284	BW25113 ***ΔsapD***	Keio collection
JW5967	BW25113 ***ΔsgcB***	Keio collection
JW2598	BW25113 ***ΔsmpA*** **(*bamE*)**	Keio collection
JW5962	BW25113 ***Δsra***	Keio collection

**Strain**	**Genotype**	**Source**
JW2847	BW25113 ***ΔssnA***	Keio collection
JW0919	BW25113 ***ΔssuA***	Keio collection
JW5738	BW25113 ***ΔsugE* (*gdx*)**	Keio collection
JW0622	BW25113 ***ΔtatE***	Keio collection
JW0067	BW25113 ***ΔtbpA* (*thiB*)**	Keio collection
LBB925	LBB451 ***tolC::Tn10***	In-house strain
JW4154	BW25113 ***ΔulaD***	Keio collection
JW2483	BW25113 ***Δupp***	Keio collection
JW3991	BW25113 ***ΔxylE***	Keio collection
JW0222	BW25113 ***ΔyafN***	Keio collection
JW0279	BW25113 ***ΔyagS* (*paoB*)**	Keio collection
JW0445	BW25113 ***ΔybaA***	Keio collection
JW0696	BW25113 ***ΔybfD***	Keio collection
JW0725	BW25113 ***ΔybgE***	Keio collection
JW0780	BW25113 ***ΔybiH* (*cecR*)**	Keio collection
JW1015	BW25113 ***ΔycdU***	Keio collection
JW1271	BW25113 ***ΔyciS* (*lapA*)**	Keio collection
JW5198	BW25113 ***ΔyciX***	Keio collection
JW5252	BW25113 ***ΔydfO***	Keio collection
JW1859	BW25113 ***ΔyecO* (*cmoA*)**	Keio collection
JW3253	BW25113 ***ΔyhdL* (*arfA*)**	Keio collection
JW3750	BW25113 ***ΔyifN***	Keio collection
JW5756	BW25113 ***ΔyjgK* (*tabA*)**	Keio collection
JW4310	BW25113 ***ΔyjiW* (*symE*)**	Keio collection
JW4341	BW25113 ***ΔyjjV***	Keio collection
JW5245	BW25113 ***ΔyneE***	Keio collection
JW4168	BW25113 ***ΔytfF***	Keio collection

Table S6. Remaining 54 candidate strains after re-imaging of initial candidates and poorly growing strains. [a] The SMG3 strain is a variant of the MG1655 wildtype. [b] The *recA306* notation refers to the *Δ(srl-recA)306* mutation, which only leaves a small portion of the *recA* gene and can be considered a deletion of *recA* (1–3).

Strain	Genotype	Source
JW2201	BW25113 ***Δada***	Keio collection (34)
JW1228	BW25113 ***ΔadhE***	Keio collection
JW4047	BW25113 ***ΔalsC***	Keio collection
JW3713	BW25113 ***ΔatpH***	Keio collection
JW0865	BW25113 ***ΔclpS***	Keio collection
JW3905	BW25113 ***ΔcytR***	Keio collection
HI1733	SC1148 ***dpiA*::*Kan***	In-house strain; (18)
HI1734	SC1148 ***dpiB*::*Kan***	In-house strain; (18)
JW3509	BW25113 ***ΔdppF***	Keio collection
JW3228	BW25113 ***ΔdusB***	Keio collection
JW1506	BW25113 ***Δego* (*lsrA*)**	Keio collection
JW1077	BW25113 ***ΔfabH***	Keio collection
WM2016	CSH26 ***fis*::*Kan***	In-house strain; (19)
JW1328	BW25113 ***Δfnr***	Keio collection
JW4112	BW25113 ***ΔfrdD***	Keio collection
JW1950	BW25113 ***ΔhchA***	Keio collection
KS1115	SMG3[a] $\boldsymbol{hda_{F85V}}$	In-house strain
JW4130	BW25113 ***Δhfq***	Keio collection
JW2470	BW25113 ***ΔhyfE***	Keio collection
JW0895	BW25113 ***ΔihfB***	Keio collection
JW3313	BW25113 ***ΔkefB***	Keio collection
JW1041	BW25113 ***ΔlpxL***	Keio collection
JW3573	BW25113 ***ΔmtlA***	Keio collection
JW5875	BW25113 ***ΔnuoB***	Keio collection
STL7742	MG1655 ***obgE*::*Tn5***	In-house strain; (2)
STL9253	MG1655 ***recA306*[b]::*Tn10***	In-house strain; (1–3)

Strain	Genotype	Source
ALS972	MG1655 ***recA938*::*Cam***	In-house strain; (32)
JW2788	BW25113 ***ΔrecB***	Keio collection
JW2790	BW25113 ***ΔrecC***	Keio collection
JW2787	BW25113 ***ΔrecD***	Keio collection
JW3677	BW25113 ***ΔrecF***	Keio collection
JW5855	BW25113 ***ΔrecQ***	Keio collection
JW0461	BW25113 ***ΔrecR***	Keio collection
JW1707	BW25113 ***ΔrpmI***	Keio collection
JW0387	BW25113 ***ΔsbcC***	Keio collection
JW0388	BW25113 ***ΔsbcD***	Keio collection
JW5404	BW25113 ***ΔsseB***	Keio collection
JW0622	BW25113 ***ΔtatE***	Keio collection
JW0067	BW25113 ***ΔtbpA*** **(*thiB*)**	Keio collection
JW1425	BW25113 ***ΔtehA***	Keio collection
JW0979	BW25113 ***ΔtorT***	Keio collection
JW0369	BW25113 ***ΔyaiW***	Keio collection
JW0370	BW25113 ***ΔyaiY***	Keio collection
JW1527	BW25113 ***ΔydeE***	Keio collection
JW1530	BW25113 ***ΔydeJ***	Keio collection
JW3255	BW25113 ***ΔyhdN***	Keio collection
JW3899	BW25113 ***ΔyiiU*** **(*zapB*)**	Keio collection
JW4018	BW25113 ***ΔyjbR***	Keio collection
JW4026	BW25113 ***ΔyjcE***	Keio collection
JW4042	BW25113 ***ΔyjcQ*** **(*mdtO*)**	Keio collection
JW4310	BW25113 ***ΔyjiW*** **(*symE*)**	Keio collection
JW4365	BW25113 ***ΔyjjY***	Keio collection
JW5244	BW25113 ***ΔyneL***	Keio collection
JW4167	BW25113 ***ΔytfE***	Keio collection

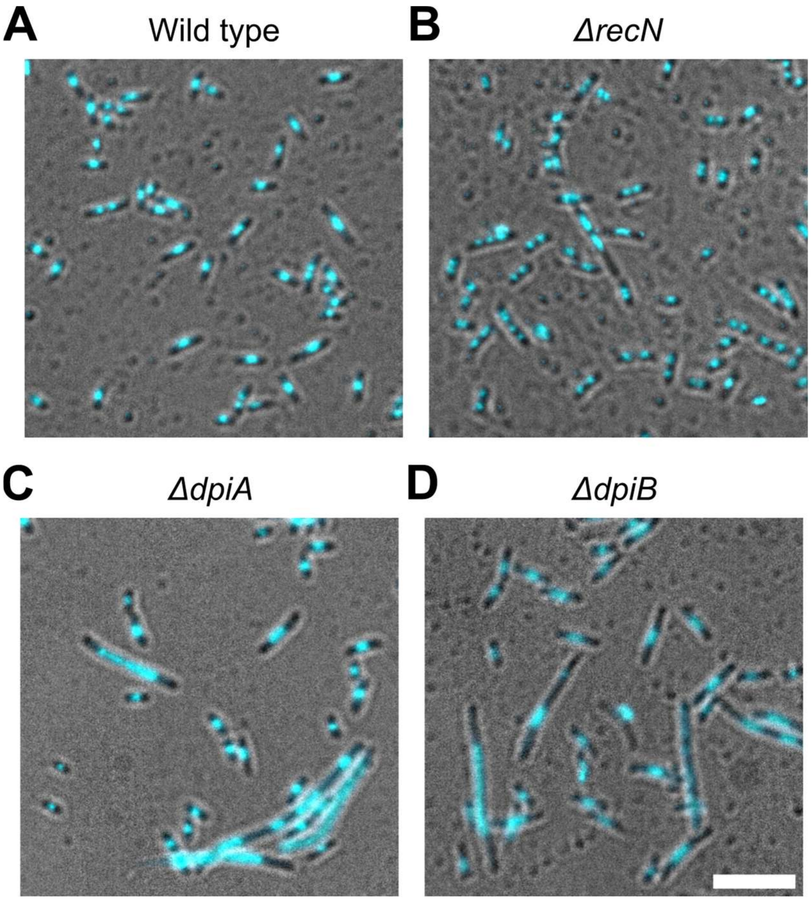

Figure S2. Example images of the *ΔdpiA* and *ΔdpiB* hit strains showing distinctive phenotypes observed in the screening's high-content imaging after 15-20 min exposure to 10 µg/mL CIP. (**A**) Wild type and (**B**) *ΔrecN* served as control strains in the screening. (**C**) *ΔdpiA* and (**D**) *ΔdpiB* strains show impaired DNA supercompaction only in a subpopulation of filamenting cells. All images are shown at the same scale; scale bar is 10 µm.

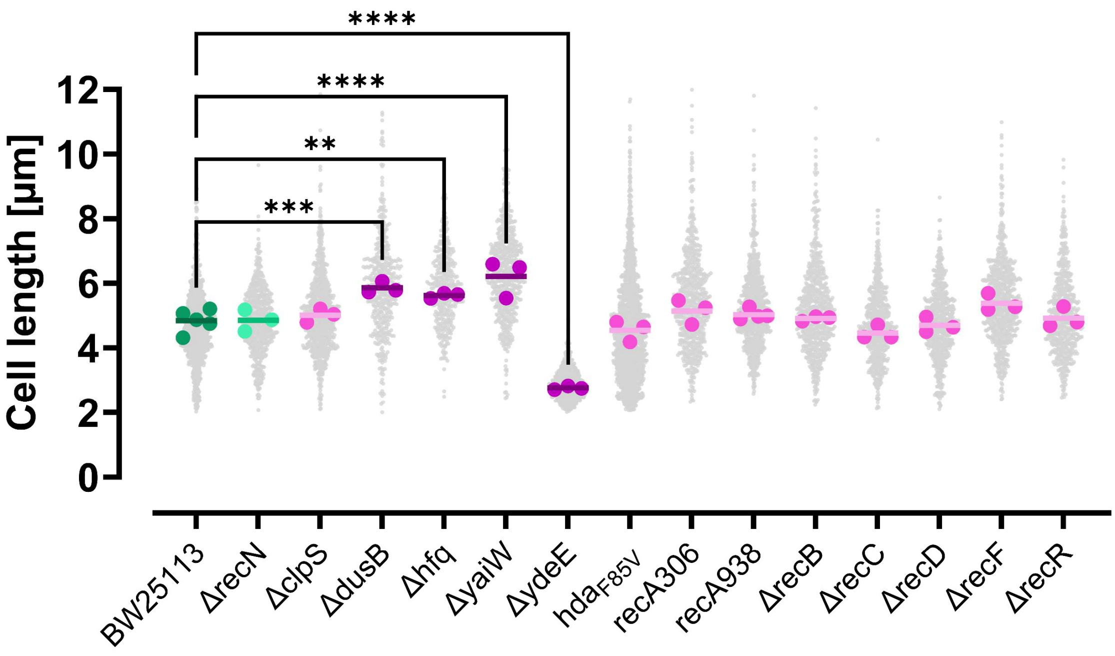

Figure S3. Cell length distributions among hit strains after 20 min exposure to 10 µg/mL CIP. Cell lengths were measured from the same images analyzed in Fig. 3, captured with live-cell spinning disk microscopy. Lines represent the mean of 3–5 biological replicates (229–1551 cells analyzed per strain per replicate). Colored dots indicate mean cell lengths from individual replicates, while small gray dots (background) show the distribution of individual cell lengths across all replicates. Wild type is shown in dark green; *ΔrecN* in light green. For strains with cell lengths significantly different from wild type, the colored dots are shown in dark magenta; for others they are shown in light magenta. Ordinary one-way ANOVA with Dunnett correction was used for statistical comparisons; only significant differences are annotated. ***P*≤0.01; ****P*≤0.001; *****P*≤0.0001.

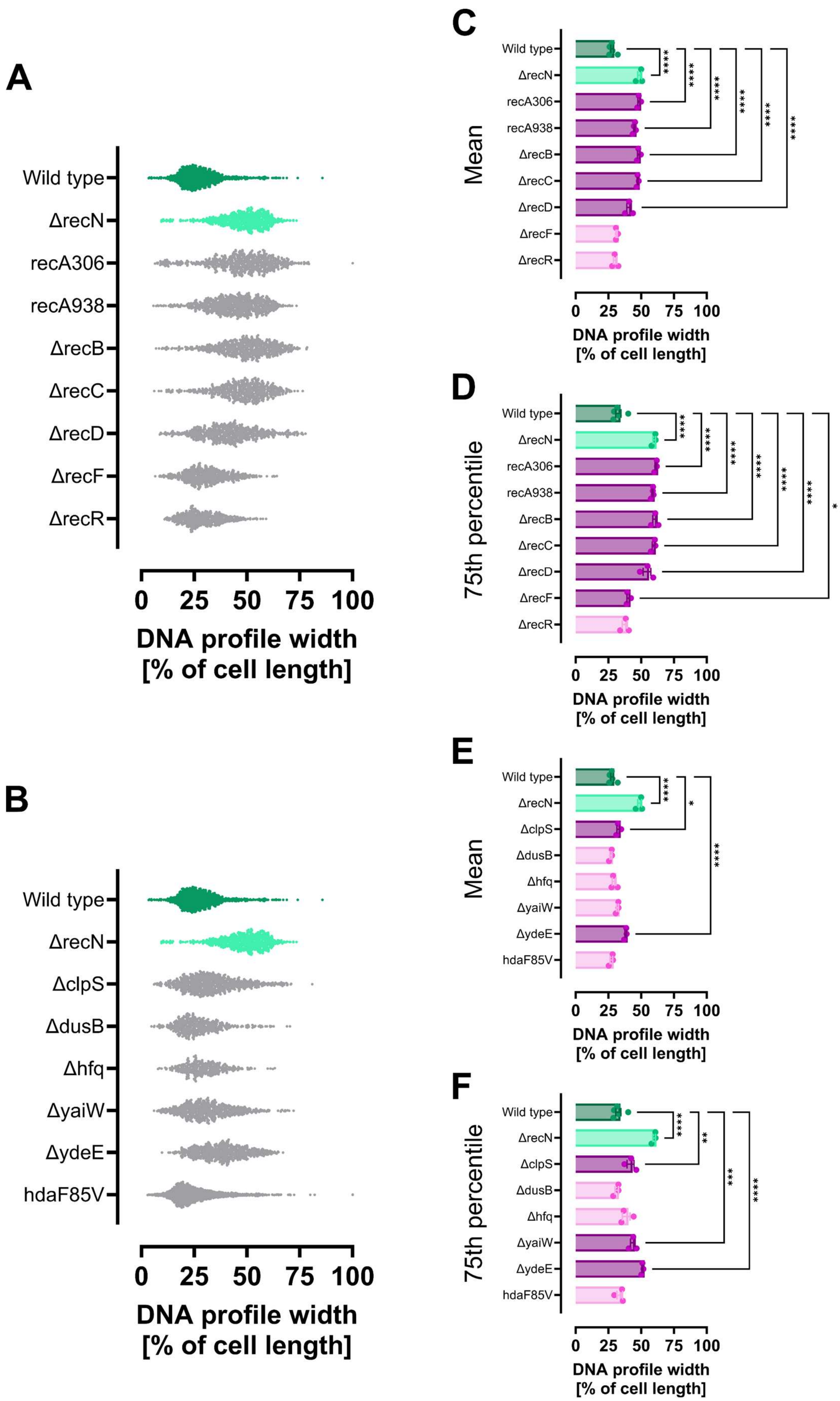

Figure S4 caption on next page.

Figure S4. Alternative metrics of DNA compaction in hit strains after 20 min exposure to 10 µg/mL CIP based on single-cell DNA profile widths (see Fig. 3). DNA profile widths were calculated for individual cells (229–1551 cells per replicate per strain) from the same images analyzed in Fig. 3, captured with live-cell spinning disk microscopy. (**A** and **B**) Distributions of single-cell DNA profile widths across all replicates. Wild type is shown in dark green; *ΔrecN* in light green; hit strains in gray. As alternative metrics for compaction, (**C** and **E**) the mean and (**D** and **F**) the 75th percentile DNA profile widths from (A) and (B) were compared for hit strains versus wild type. Strains with deletions of recombinational repair genes are shown in (A, C, and D), while strains with deletions or mutations of novel genes not previously associated with DNA compaction or repair are shown in (B, E, and F). Columns represent the mean metric values of 3–5 biological replicates; dots indicate replicate values; error bars show standard error of the mean (SEM). Wild type is shown in dark green; *ΔrecN* in light green. Strains with values significantly different from wild type are shown in dark magenta; others in light magenta. Ordinary one-way ANOVA with Dunnett correction was used for statistical comparisons; only significant differences are annotated. *$P \leq 0.05$; **$P \leq 0.01$; ***$P \leq 0.001$; ****$P \leq 0.0001$.

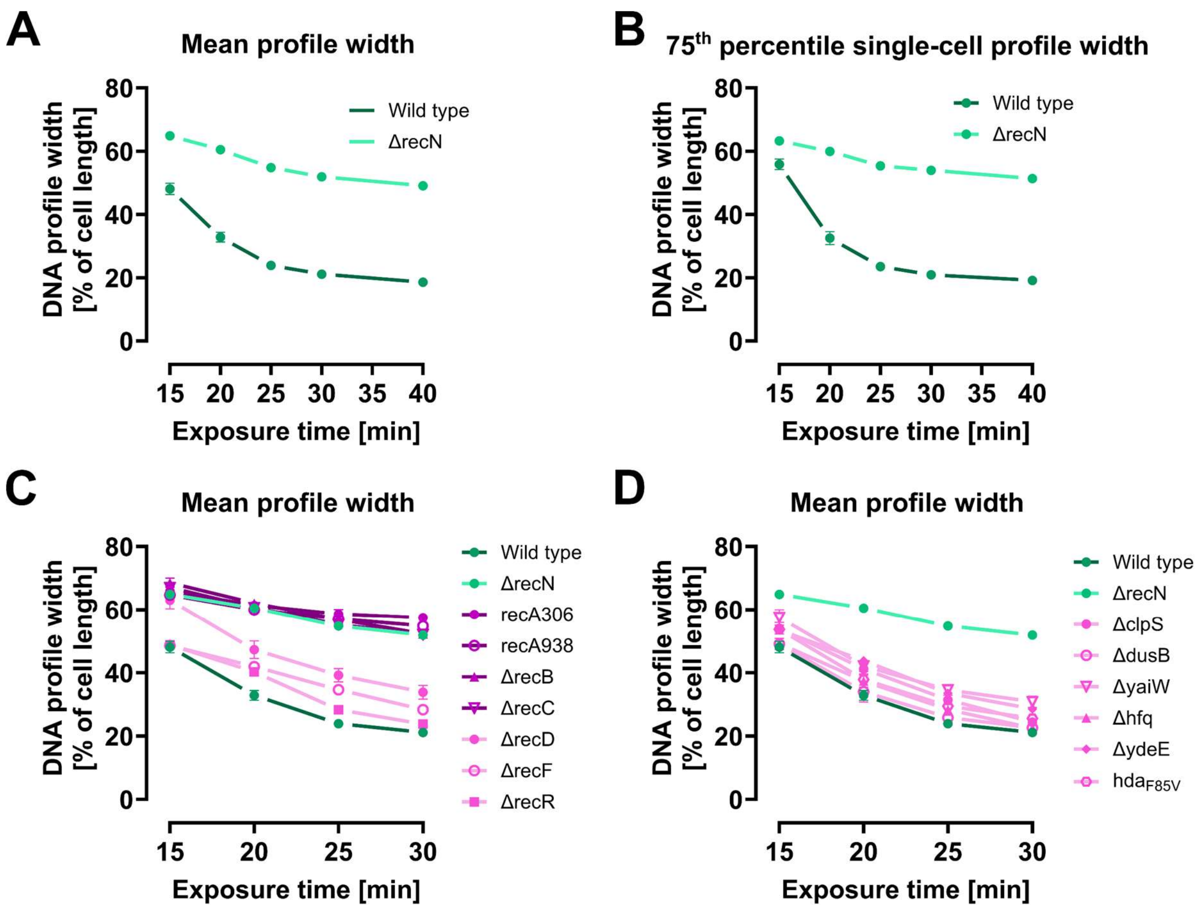

Figure S5. Comparison of DNA compaction metrics derived from mean and single-cell DNA profile widths. Both metrics show similar compaction dynamics. DNA profile widths were calculated from the same image series analyzed in Fig. 3 and Supplementary Fig. S4, captured with live-cell spinning disk microscopy. (**A** and **B**) DNA compaction metrics calculated for wild type and *ΔrecN* strains over time using two approaches: (A) the mean DNA profile width derived from the averaged DNA profile for each replicate, and (B) the 75th percentile of single-cell DNA profile widths. Measurements were obtained at 20, 25, 30, and 40 min after exposure to 10 µg/mL CIP. (**C** and **D**) Time-dependent DNA compaction of hit strains compared with wild type and *ΔrecN* using the mean DNA profile width metric. Measurements were obtained at 15, 20, 25, and 30 min after CIP exposure. Strains with deletions of recombinational repair genes are shown in (C), while those with deletions or mutations of novel genes not previously associated with DNA compaction or repair are shown in (D). Dots represent mean values from 3–5 biological replicates; error bars show SEM. Wild type is shown in dark green; *ΔrecN* in light green. Strains with profile widths similar to *ΔrecN* over time are shown in dark magenta; others in light magenta.

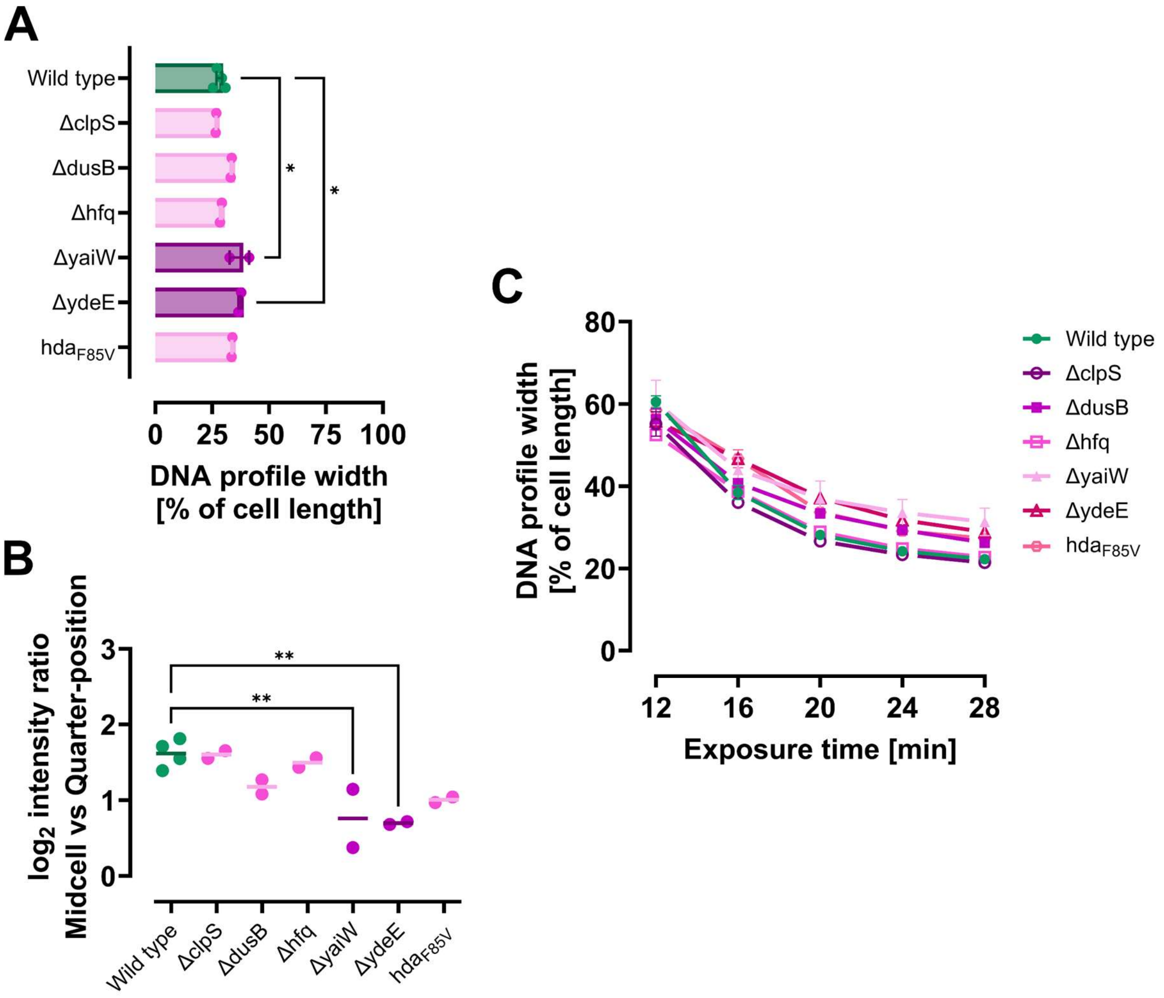

Figure S6. Comparison of DNA compaction metrics for novel hit strains carrying the pSOS plasmid for GFP-RecN expression versus wild type after 10 µg/mL CIP exposure. Measurements are derived from the same image series analyzed in Fig. 4, captured with live-cell spinning disk microscopy. (**A**) Mean DNA profile widths for hit strains compared with wild type after 20 min CIP exposure, calculated as described in Fig. 3. (**B**) Log_2-transformed ratio of fluorescence intensities between midcell (50% of cell length) and the mean of the quarter-positions (25% and 75% of cell length) after 20 min CIP exposure. Columns and lines represent means from 2–4 biological replicates; dots indicate individual replicate values; error bars show SEM. Wild type is shown in dark green. Strains with significantly different values from wild type are shown in dark magenta; others in light magenta. (**C**) Time-dependent DNA compaction of hit strains compared with wild type using the mean DNA profile width metric, measured at 12, 16, 20, 24, and 28 min after CIP exposure. Dots represent mean values from 2–4 biological replicates; error bars show SEM. Ordinary one-way ANOVA with Dunnett correction was used for statistical comparisons; only significant differences are annotated. *$P \leq 0.05$; **$P \leq 0.01$.

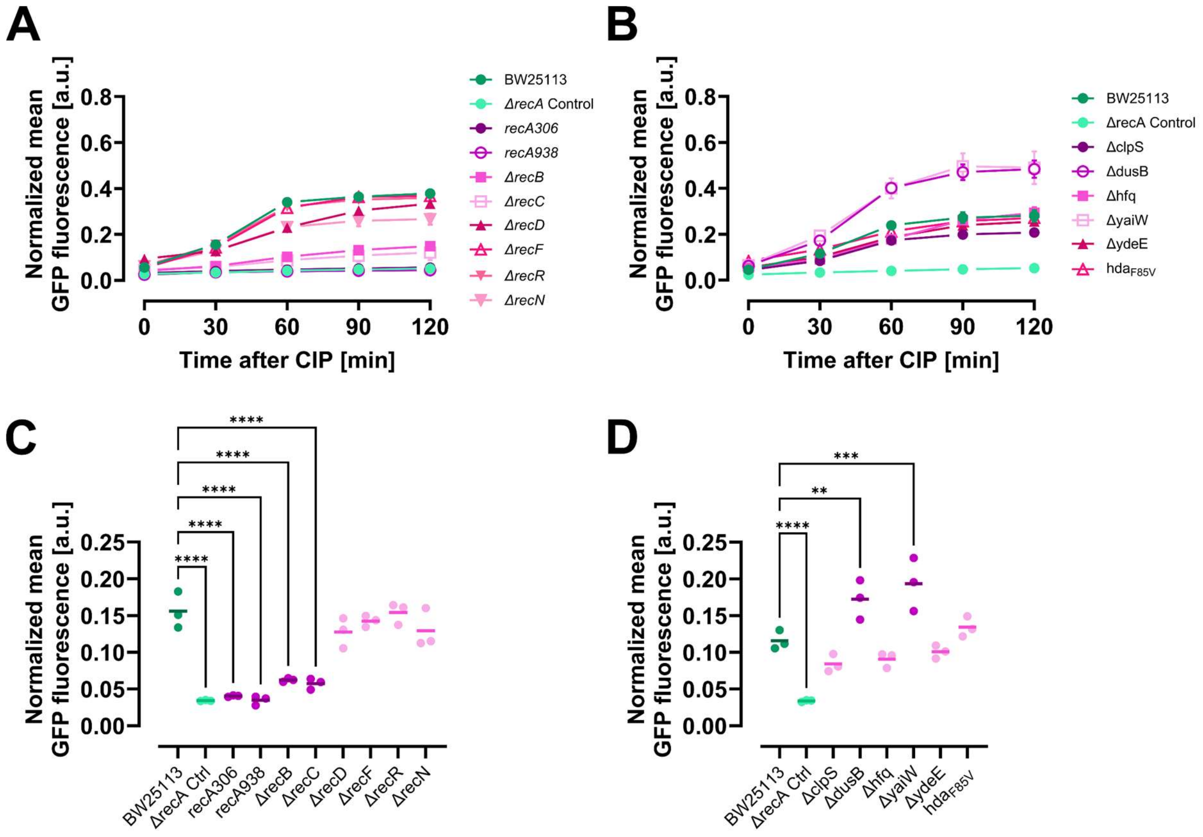

Figure S7. Supplementary quantifications of SOS response activity in hit strains after exposure to 10 µg/mL CIP. SOS response activity was quantified using the same *lexA* promoter-regulated GFP reporter assay data described in Fig. 5. (**A** and **B**) Time-dependent SOS activity measured at 30-min intervals from baseline (0 min) to 120 min after CIP exposure. (**C** and **D**) SOS activity measured 30 min after CIP exposure. The assay was performed separately for strains with mutations or deletions of recombinational repair genes (A and C), and novel genes not previously associated with DNA compaction or repair (B and D). A *ΔrecA* strain from the Keio collection (JW2669) served as the negative control. (A and B) Symbols with connecting lines represent means from three biological replicates; error bars indicate SEM. (C and D) Lines represent means from three biological replicates; dots indicate individual replicate means. Wild type (BW25113) is presented in dark green, *ΔrecA* in light green. Strains with activities significantly different from the corresponding wild type are shown in dark magenta; others in light magenta. Ordinary one-way ANOVA with Dunnett correction was used for statistical comparisons; only significant differences are annotated. $^{**}P \le 0.01$; $^{***}P \le 0.001$; $^{****}P \le 0.0001$.

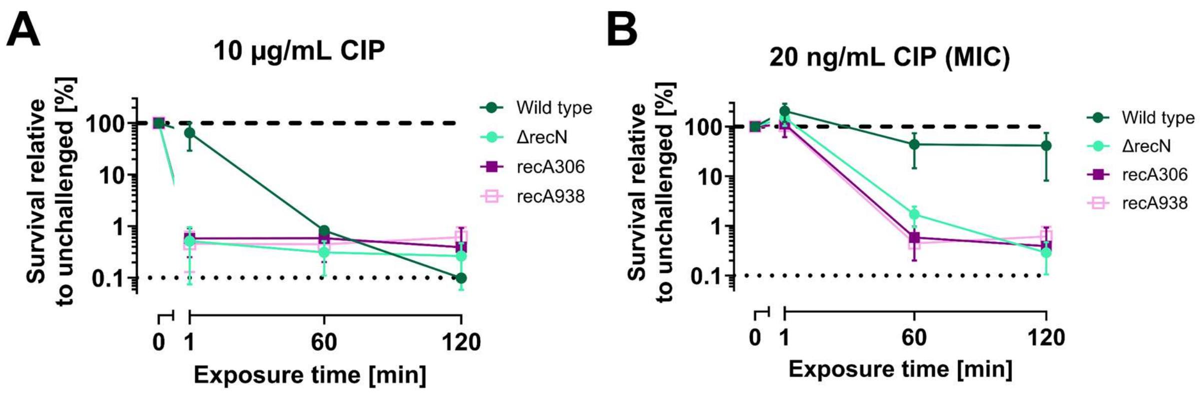

Figure S8. Time-dependent survival of *recA* deletion hit strains after CIP exposure versus wild type and *ΔrecN*. Survival assays were performed as described for Fig. 6. Relative survival was measured for wild type, *ΔrecN*, *recA306*, and *recA938* strains at 1, 60, and 120 min after exposure to CIP at a dose of (**A**) 10 µg/mL or (**B**) 20 ng/mL (MIC). Symbols with connecting lines represent means from three biological replicates; error bars indicate standard deviation. Thick dashed lines indicate survival of unchallenged parallels; thin dotted lines mark the assay's detection limit.

Table S7. Estimates for fixed effects from a linear mixed-effects model assessing time-dependent survival after exposure to CIP for wild type, *ΔrecN*, *recA306*, and *recA938* strains. Relative survival values (Supplementary Fig. S8) were log_{10}-transformed and modelled with exposure time (minutes), CIP dose (10 µg/mL vs MIC), and strain (wild type as reference) as fixed effects, and biological replicates as random intercepts. The (Intercept) estimate represents the log_{10} relative survival of the wild type prior to CIP exposure. The estimate for CIP exposure time indicates the change in log_{10} relative survival per minute of exposure, while estimates for each deletion strain reflect their difference in log_{10} relative survival from the wild type. The estimate for CIP dose represents the difference in log_{10} relative survival between the two CIP doses. A change of -1 in log_{10} relative survival corresponds to a 10-fold (90%) reduction in survival. 95% confidence intervals are shown for each estimate, along with *P*-values derived from testing whether the fixed effects differ from zero—i.e. whether survival changes with exposure time or increased CIP dose, and whether deletion strain sensitivities differ from the wild type. *P*-values and confidence intervals for deletion effects relative to wild type were adjusted with Dunnett correction for multiple comparisons.

**Fixed effect**	**Estimate**	**95% Confidence interval**	***P* value**
(Intercept)	2.298	[1.890, 2.707]	<0.0001
CIP exposure time	-0.013	[-0.016, -0.009]	<0.0001
CIP dose, 10 µg/mL vs MIC	-1.074	[-1.394, -0.755]	<0.0001
recA306 vs Wild type	-1.082	[-1.741, -0.424]	0.0037
recA938 vs Wild type	-0.943	[-1.601, -0.284]	0.0083
ΔrecN vs Wild type	-0.987	[-1.646, -0.328]	0.0064

Table S8. Estimates for fixed effects from a linear mixed-effects model assessing time-dependent survival after exposure to 10 µg/mL CIP for all hit strains except those with *recA* deletions. Relative survival values (Fig. 6A and B) were log_{10}-transformed and modelled with exposure time (minutes) and strain (wild type as reference) as fixed effects, and biological replicates as random intercepts. The (Intercept) estimate represents the log_{10} relative survival of the wild type prior to CIP exposure. The estimate for CIP exposure time indicates the change in log_{10} relative survival per minute of exposure, while estimates for each deletion strain reflect their difference in log_{10} relative survival from the wild type. A change of -1 in log_{10} relative survival corresponds to a 10-fold (90%) reduction in survival. 95% confidence intervals are shown for each estimate, along with *P*-values derived from testing whether the fixed effects differ from zero—i.e. whether survival changes with exposure time and whether deletion strain sensitivities differ from the wild type. *P*-values and confidence intervals for deletion effects relative to wild type were adjusted with Dunnett correction for multiple comparisons.

**Fixed effect**	**Estimate**	**95% Confidence interval**	***P*-value**
(Intercept)	1.343	[1.191, 1.494]	<0.0001
CIP exposure time	-0.021	[-0.023, -0.019]	<0.0001
ΔclpS vs Wild type	-0.350	[-0.821, 0.122]	0.2412
ΔdusB vs Wild type	-0.423	[-0.894, 0.048]	0.0988
hda$_{F85V}$ vs Wild type	-0.873	[-1.293, -0.453]	<0.0001
Δhfq vs Wild type	0.350	[-0.121, 0.821]	0.2406
ΔrecB vs Wild type	-1.222	[-1.693, -0.751]	<0.0001
ΔrecC vs Wild type	-1.374	[-1.845, -0.903]	<0.0001
ΔrecD vs Wild type	0.587	[0.116, 1.058]	0.0080
ΔrecF vs Wild type	0.853	[0.429, 1.278]	<0.0001
ΔrecR vs Wild type	-0.253	[-0.678, 0.171]	0.4658
ΔyaiW vs Wild type	-0.365	[-0.836, 0.106]	0.2030
ΔydeE vs Wild type	0.565	[0.094, 1.036]	0.0115

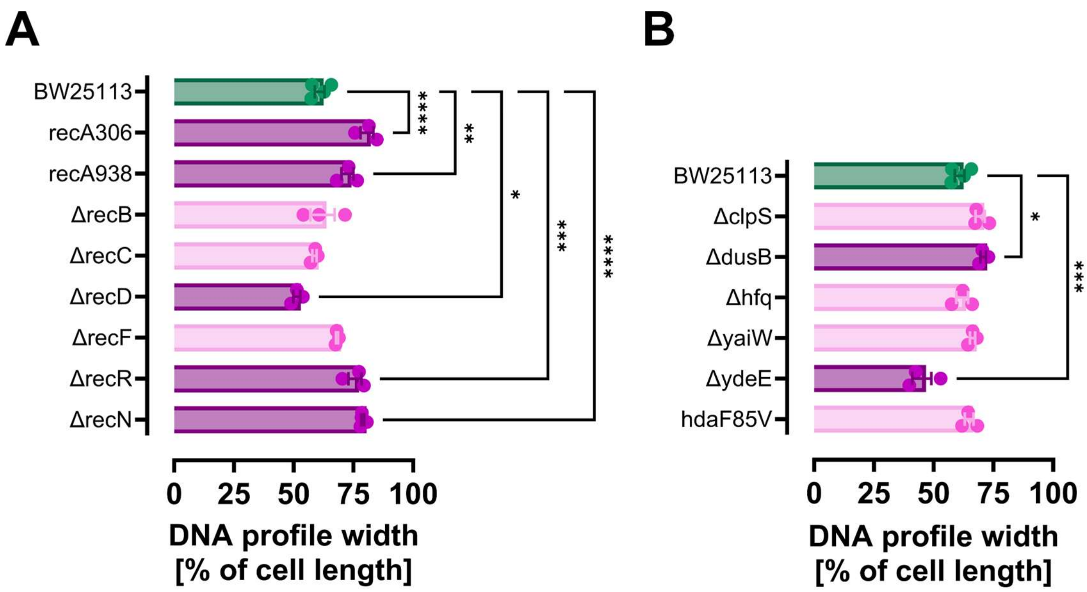

Figure S9. DNA compaction analysis for hit strains following UV exposure. Strains were irradiated with 5 J/m^2 UV and allowed a 15-min recovery, before fixing, staining with Hoechst 33258, and imaging by fluorescence microscopy (similar approach to the validation experiments for the screening candidate strains). (**A** and **B**) DNA compaction was quantified by measuring DNA profile widths as described in Fig. 3. Hit strains with deletions of recombinational repair genes, including the *ΔrecN* strain, are shown in (A), while hit strains with deletions or mutations of novel genes not previously associated with DNA compaction or repair are shown in (B). Columns represent means from 3-4 biological replicates; dots show individual replicate values; error bars indicate SEM. The wild type is shown in dark green. Strains with values significantly different from wild type are shown in dark magenta; others in light magenta. Ordinary one-way ANOVA with Dunnett correction was used for statistical comparisons; only significant differences are annotated. *P≤0.05; **P≤0.01; ***P≤0.001; ****P≤0.0001.

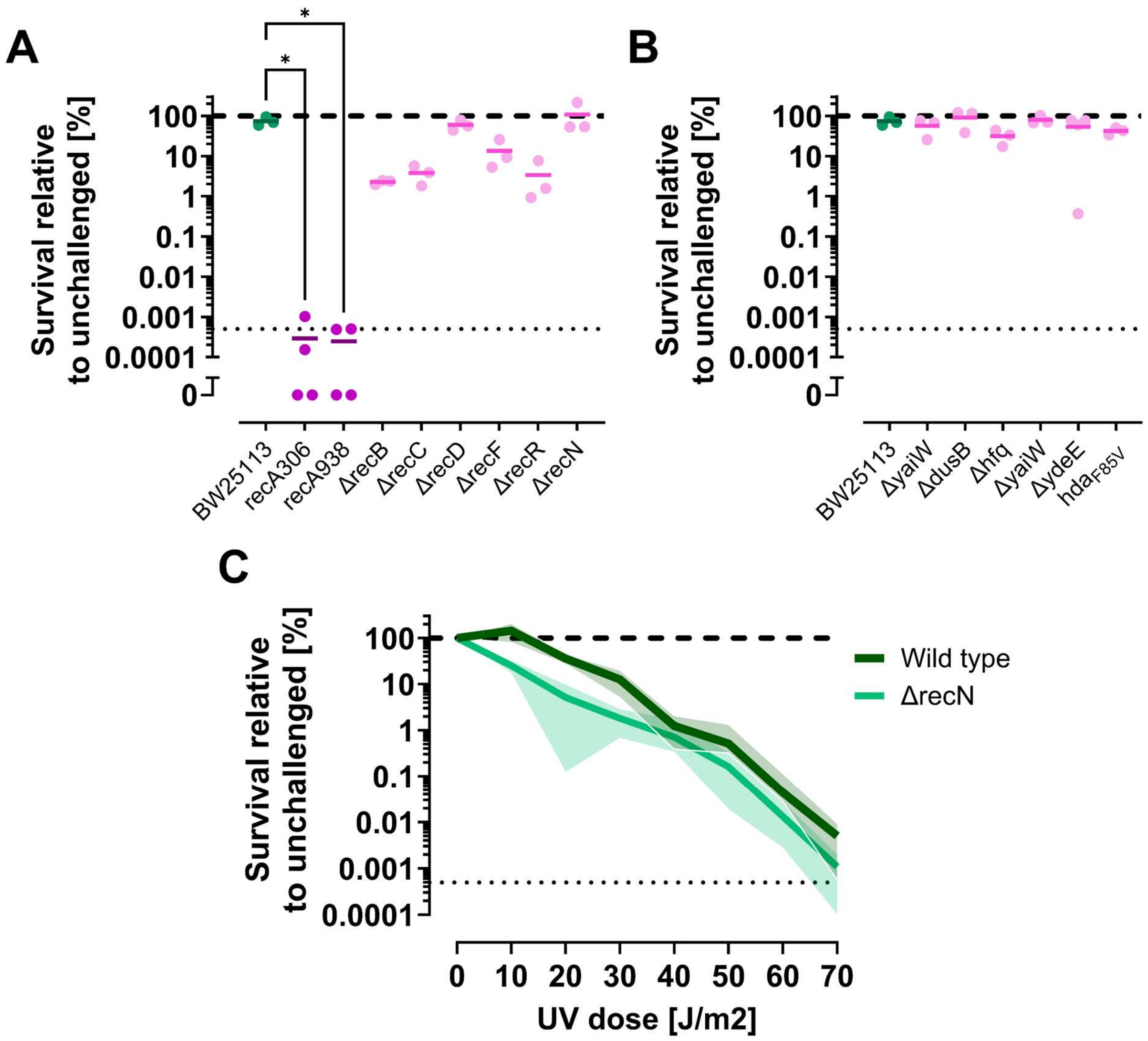

Figure S10. UV sensitivity varies among hit strains, while the *ΔrecN* strain (JW5416) displays sensitivity similar to wild type (BW25113). (**A** and **B**) Survival after 5 J/m^2 UV irradiation for hit strains with deletions of recombinational repair genes, including *ΔrecN*, is shown in (A), while hit strains with mutations or deletions of novel genes not previously associated with DNA compaction or repair are shown in (B). (**C**) Dose-dependent survival of *ΔrecN* compared with wild type across UV doses ranging from 10–70 J/m^2. Relative survival was calculated from colony forming units (CFU) per mL by normalization to an unchallenged parallel culture originating from the same population before UV irradiation. Solid lines represent means from 3–4 biological replicates; dots indicate individual replicate values; shaded regions indicate standard deviation. Wild type is shown in dark green. Strains with UV sensitivity significantly different from wild type are shown in dark magenta; others in light magenta. Thick dashed lines show survival of unchallenged parallels; thin dotted lines mark the assay's detection limit. Ordinary one-way ANOVA with Dunnett correction was used for statistical comparisons; only significant differences are annotated. *$P \leq 0.05$.

Table S9. Estimates for fixed effects from a linear mixed-effects model assessing dose-dependent survival of the *ΔrecN* strain versus wild type for a range of UV doses. Relative survival values (Supplementary Fig. S13C) were log_{10}-transformed and modelled with UV dose (J/m^2) and strain (wild type as reference) as fixed effects, and biological replicates as random intercepts. The (Intercept) estimate represents the log_{10} relative survival of the wild type prior to UV irradiation. The estimate for UV dose indicates the change in log_{10} relative survival per J/m^2 UV irradiation, while the estimate for *ΔrecN* reflect its difference in log_{10} relative survival from the wild type. A change of -1 in log_{10} relative survival corresponds to a 10-fold (90%) reduction in survival. 95% confidence intervals are shown for each estimate, along with *P*-values derived from testing whether the fixed effects differ from zero—i.e. whether survival changes with UV dose and whether the *ΔrecN* strain's sensitivity differs from the wild type.

**Fixed effect**	**Estimate**	**95% Confidence interval**	***P* value**
(Intercept)	2.976	[2.501, 3.451]	<0.0001
UV dose	-0.077	[-0.082, -0.071]	<0.0001
ΔrecN	-0.604	[-1.244, 0.036]	0.0603

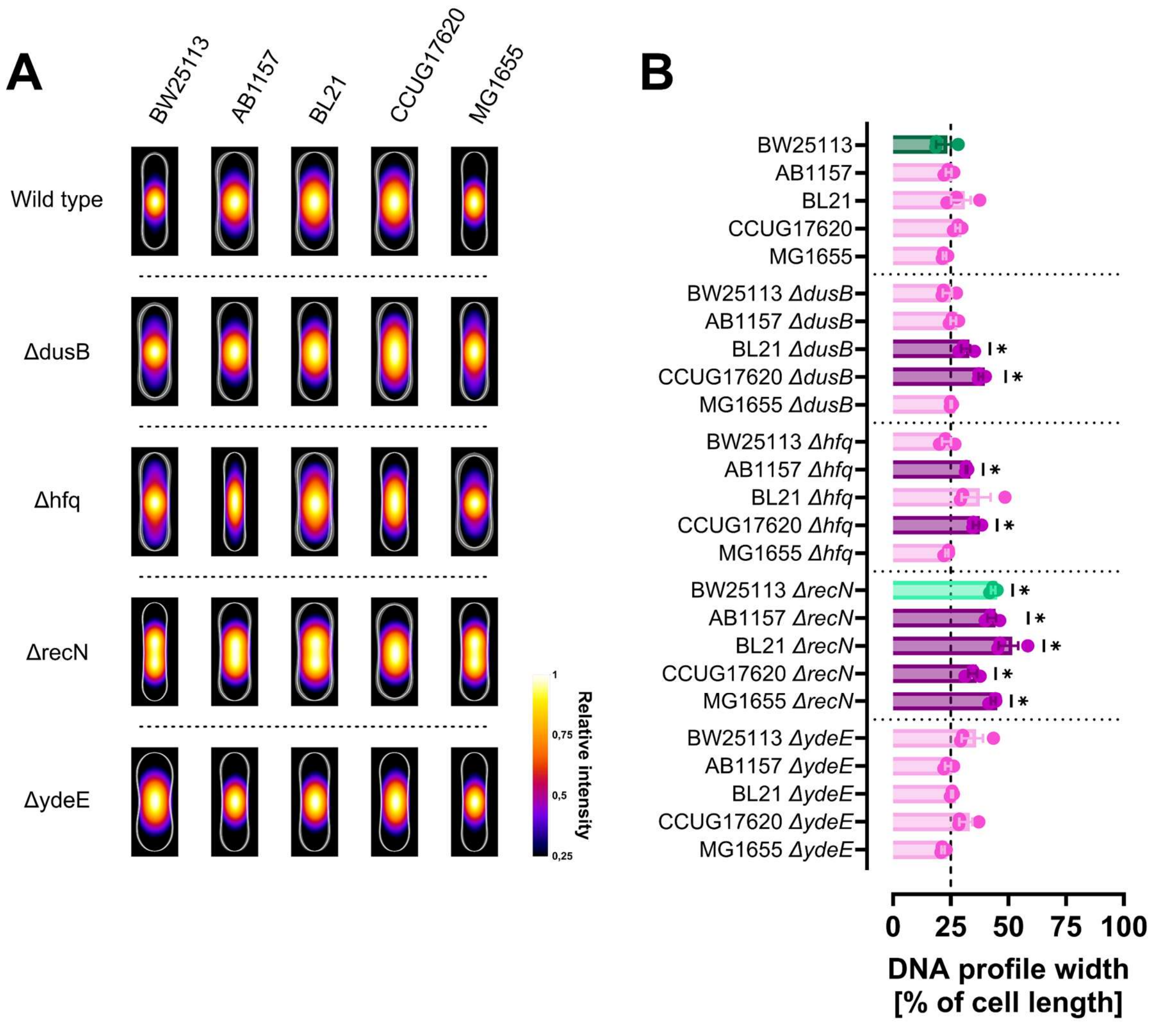

Figure S11. Influence of different *E. coli* wild-type backgrounds on DNA compaction dynamics following deletions of *dusB*, *hfq*, *recN*, or *ydeE*. Strains derived from five genetic backgrounds (BW25113, AB1157, BL21, CCUG17620, and MG1655) were exposed to 10 µg/mL CIP for 20 min, fixed, and stained with Hoechst 33258 for DNA visualization prior to high-content imaging (similar approach as used in the screening). (**A**) Heat maps showing relative intensity distributions of Hoechst 33258 within cells for representative biological replicates. (**B**) Comparison of DNA profile widths in the various strains with the expected upper limit of 25% of cell length for completed wild-type DNA supercompaction. Profile widths were quantified as described in Fig. 3. Columns represent means from three biological replicates; dots indicate individual replicate values. Wild type (BW25113) is shown in dark green; *ΔrecN* (in BW25113 background) in light green. Strains with DNA profile widths significantly wider than 25% of cell length are shown in dark magenta; others in light magenta. One-sample, one-tailed t-tests were used to test whether DNA profile widths were significantly wider than 25% of cell length; only significant differences are annotated, using a single significance level: *$P \leq 0.05$.

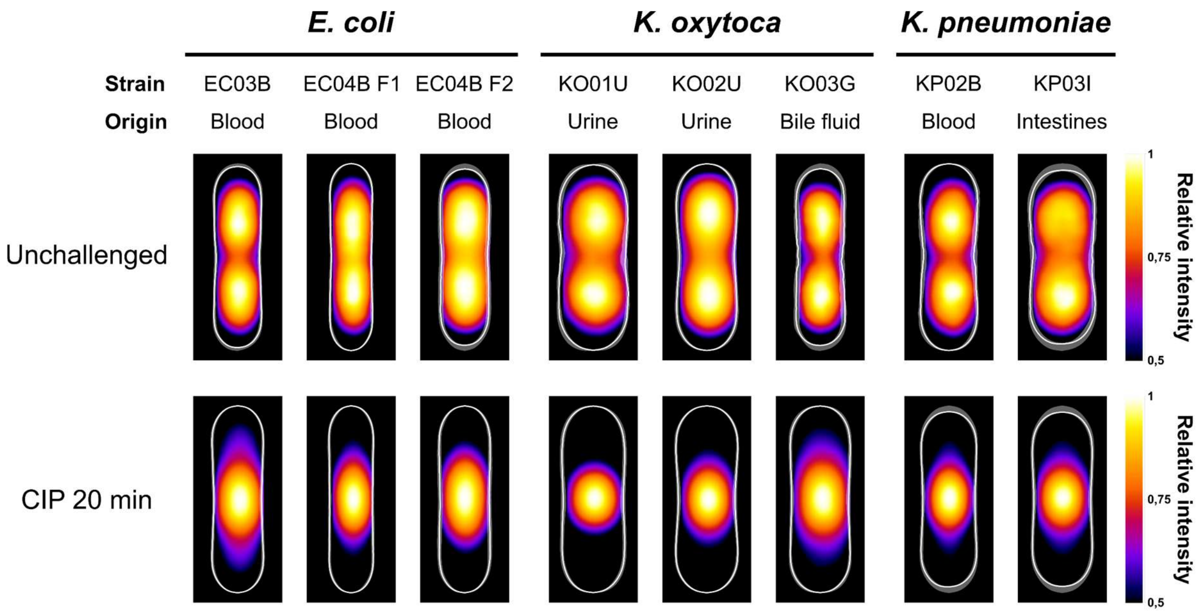

Figure S12. DNA supercompaction occurs in clinical strains following CIP exposure. Strains include *E. coli*, *K. oxytoca*, and *K. pneumoniae*, isolated from blood, urine, bile fluid, and intestines. Heat maps show relative Hoechst 33258 intensity distribution in unchallenged cells (top row) and cells exposed to 10 µg/mL CIP for 20 minutes (bottom row). Results are from representative biological replicates and are averaged from 37–1395 cells per sample. EC04B F1 and EC04B F2 are distinct strains from the same patient.

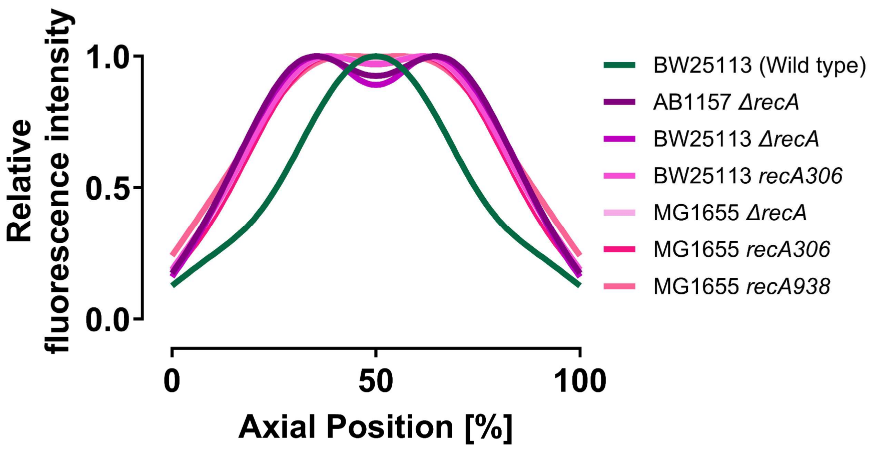

Figure S13. Comparison of DNA profiles along the cells' long axis after 20 minutes of CIP exposure (10 µg/mL) for various strains with *recA* deletions against the BW25113 wild type, used to evaluate compaction impairment in *recA* deletion strains.

REFERENCES

1. Suzuki S, Kaidow A, Meya T *et al.* Phenotypic difference between Δ(srl–recA)306 and ΔrecA::Km elucidated by next-generation sequencing combined with a long-PCR system. *J. Gen. Appl. Microbiol.* 2017;**63**:22–27. doi: 10.2323/jgam.2016.07.002.

2. Foti JJ, Schienda J, Sutera VA *et al.* A Bacterial G Protein-Mediated Response to Replication Arrest. *Mol. Cell*. 2005;**17**:549–60. doi: 10.1016/j.molcel.2005.01.012.

3. Csonka LN and Clark AJ. Deletions generated by the transposon Tn10 in the srl recA region of the Escherichia coli K-12 chromosome. *Genetics*. 1979;**93**:321–43. doi: 10.1093/genetics/93.2.321.

4. Weel-Sneve R, Kristiansen KI, Odsbu I *et al.* Single Transmembrane Peptide DinQ Modulates Membrane-Dependent Activities. *PLoS Genet.* 2013;**9**:e1003260. doi: 10.1371/journal.pgen.1003260.

5. Pedersen IB, Helgesen E, Flåtten I *et al.* SeqA structures behind Escherichia coli replication forks affect replication elongation and restart mechanisms. *Nucleic Acids Res.* 2017;**45**:6471–85. doi: 10.1093/nar/gkx263.

6. Fossum S, Søreide S and Skarstad K. Lack of SeqA focus formation, specific DNA binding and proper protein multimerization in the Escherichia coli sequestration mutant seqA2. *Mol. Microbiol.* 2003;**47**:619–32. doi: 10.1046/j.1365-2958.2003.t01-1-03329.x.

7. Singer M, Baker TA, Schnitzler G *et al.* A collection of strains containing genetically linked alternating antibiotic resistance elements for genetic mapping of Escherichia coli. *Microbiol. Rev.* 1989;**53**:1–24. doi: 10.1128/mr.53.1.1-24.1989.

8. Nichols BP, Shafiq O and Meiners V. Sequence Analysis of Tn10 Insertion Sites in a Collection of Escherichia coli Strains Used for Genetic Mapping and Strain Construction. *J. Bacteriol.* 1998;**180**:6408–11. doi: 10.1128/JB.180.23.6408-6411.1998.

9. Ozaki S, Matsuda Y, Keyamura K *et al.* A Replicase Clamp-Binding Dynamin-like Protein Promotes Colocalization of Nascent DNA Strands and Equipartitioning of Chromosomes in E. coli. *Cell Rep.* 2013;**4**:985–95. doi: 10.1016/j.celrep.2013.07.040.

10. Marinus MG, Carraway M, Frey AZ *et al.* Insertion mutations in the dam gene of Escherichia coli K-12. *Mol. Gen. Genet. MGG.* 1983;**192**:288–89. doi: 10.1007/BF00327681.

11. Parker B and Marinus MG. A simple and rapid method to obtain substitution mutations in *Escherichia coli*: isolation of a *dam* deletion/insertion mutation. *Gene*. 1988;**73**:531–35. doi: 10.1016/0378-1119(88)90517-3.

12. Boeneman K, Fossum S, Yang Y *et al.* Escherichia coli DnaA forms helical structures along the longitudinal cell axis distinct from MreB filaments. *Mol. Microbiol.* 2009;**72**:645–57. doi: 10.1111/j.1365-2958.2009.06674.x.

13. Leslie NR and Sherratt DJ. Site-specific recombination in the replication terminus region of Escherichia coli: functional replacement of dif. *EMBO J.* 1995;**14**:1561–70. doi: 10.1002/j.1460-2075.1995.tb07142.x.

14. Slominska M, Wahl A, Wegrzyn G *et al.* Degradation of mutant initiator protein DnaA204 by proteases ClpP, ClpQ and Lon is prevented when DNA is SeqA-free. *Biochem. J.* 2003;**370**:867–71. doi: 10.1042/BJ20021161.

15. Gon S, Camara JE, Klungsøyr HK *et al.* A novel regulatory mechanism couples deoxyribonucleotide synthesis and DNA replication in Escherichia coli. *EMBO J.* 2006;**25**:1137–47. doi: 10.1038/sj.emboj.7600990.

16. Feeney MA, Ke N and Beckwith J. Mutations at Several Loci Cause Increased Expression of Ribonucleotide Reductase in Escherichia coli. *J. Bacteriol.* 2012;**194**:1515–22. doi: 10.1128/jb.05989-11.

17. Sandler SJ, Samra HS and Clark AJ. Differential suppression of priA2::kan phenotypes in Escherichia coli K-12 by mutations in priA, lexA, and dnaC. *Genetics*. 1996;**143**:5–13. doi: 10.1093/genetics/143.1.5.

18. Ingmer H, Miller CA and Cohen SN. Destabilized inheritance of pSC101 and other Escherichia coli plasmids by DpiA, a novel two-component system regulator. *Mol. Microbiol.* 1998;**29**:49–59. doi: 10.1046/j.1365-2958.1998.00895.x.

19. Bates DB, Boye E, Asai T *et al.* The absence of effect of gid or mioC transcription on the initiation of chromosomal replication in Escherichia coli. *Proc. Natl. Acad. Sci.* 1997;**94**:12497–502. doi: 10.1073/pnas.94.23.12497.

20. Khodursky AB, Zechiedrich EL and Cozzarelli NR. Topoisomerase IV is a target of quinolones in Escherichia coli. *Proc. Natl. Acad. Sci.* 1995;**92**:11801–05. doi: 10.1073/pnas.92.25.11801.

21. Morgan-Linnell SK and Zechiedrich L. Contributions of the Combined Effects of Topoisomerase Mutations toward Fluoroquinolone Resistance in Escherichia coli. *Antimicrob. Agents Chemother.* 2007;**51**:4205–08. doi: 10.1128/aac.00647-07.

22. Johnsen L, Weigel C, von Kries J *et al.* A novel DNA gyrase inhibitor rescues Escherichia coli dnaAcos mutant cells from lethal hyperinitiation. *J. Antimicrob. Chemother.* 2010;**65**:924–30. doi: 10.1093/jac/dkq071.

23. Løbner-Olesen A and von Freiesleben U. Chromosomal replication incompatibility in Dam methyltransferase deficient Escherichia coli cells. *EMBO J.* 1996;**15**:5999–6008. doi: 10.1002/j.1460-2075.1996.tb00986.x.

24. Weitao T, Nordström K and Dasgupta S. Mutual suppression of mukB and seqA phenotypes might arise from their opposing influences on the Escherichia coli nucleoid structure. *Mol. Microbiol.* 1999;**34**:157–68. doi: 10.1046/j.1365-2958.1999.01589.x.

25. Elez M, Murray AW, Bi L-J *et al.* Seeing Mutations in Living Cells. *Curr. Biol.* 2010;**20**:1432–37. doi: 10.1016/j.cub.2010.06.071.

26. Nowosielska A, Calmann MA, Zdraveski Z *et al.* Spontaneous and cisplatin-induced recombination in *Escherichia coli*. *DNA Repair*. 2004;**3**:719–28. doi: 10.1016/j.dnarep.2004.02.009.

27. Elez M, Radman M and Matic I. Stoichiometry of MutS and MutL at unrepaired mismatches in vivo suggests a mechanism of repair. *Nucleic Acids Res.* 2012;**40**:3929–38. doi: 10.1093/nar/gkr1298.

28. Nowosielska A and Marinus MG. Cisplatin induces DNA double-strand break formation in *Escherichia coli dam* mutants. *DNA Repair*. 2005;**4**:773–81. doi: 10.1016/j.dnarep.2005.03.006.

29. Siegel EC, Wain SL, Meltzer SF *et al.* Mutator mutations in *Escherichia coli* induced by the insertion of phage Mu and the transposable resistance elements Tn5 and Tn10. *Mutat. Res. Mol. Mech. Mutagen.* 1982;**93**:25–33. doi: 10.1016/0027-5107(82)90122-1.

30. Masai H, Asai T, Kubota Y *et al.* Escherichia coli PriA protein is essential for inducible and constitutive stable DNA replication. *EMBO J.* 1994;**13**:5338–45. doi: 10.1002/j.1460-2075.1994.tb06868.x.

31. Ishihara K, Ishihara M, Takazoe I *et al.* Cloning and expression of the aspartate carbamoyltransferase gene from Treponema denticola. *Appl. Environ. Microbiol.* 1992;**58**:3399–403. doi: 10.1128/aem.58.10.3399-3403.1992.

32. Zyskind JW, Svitil AL, Stine WB *et al.* RecA protein of Escherichia coli and chromosome partitioning. *Mol. Microbiol.* 1992;**6**:2525–37. doi: 10.1111/j.1365-2958.1992.tb01429.x.

33. Minvielle MJ, Eguren K and Melander C. Highly Active Modulators of Indole Signaling Alter Pathogenic Behaviors in Gram-Negative and Gram-Positive Bacteria. *Chem. Weinh. Bergstr. Ger.* 2013;**19**:17595–602. doi: 10.1002/chem.201303510.

34. Baba T, Ara T, Hasegawa M *et al.* Construction of Escherichia coli K-12 in-frame, single-gene knockout mutants: the Keio collection. *Mol. Syst. Biol.* 2006;**2**:2006.0008. doi: 10.1038/msb4100050.

35. Torheim NK, Boye E, Løbner-Olesen A *et al.* The Escherichia coli SeqA protein destabilizes mutant DnaA204 protein. *Mol. Microbiol.* 2000;**37**:629–38. doi: 10.1046/j.1365-2958.2000.02031.x.

36. von Freiesleben U, Rasmussen KV and Schaechter M. SeqA limits DnaA activity in replication from oriC in Escherichia coli. *Mol. Microbiol.* 1994;**14**:763–72. doi: 10.1111/j.1365-2958.1994.tb01313.x.

37. Godoy VG, Jarosz DF, Walker FL *et al.* Y-family DNA polymerases respond to DNA damage-independent inhibition of replication fork progression. *EMBO J.* 2006;**25**:868–79. doi: 10.1038/sj.emboj.7600986.

38. Colloms SD, Sykora P, Szatmari G *et al.* Recombination at ColE1 cer requires the Escherichia coli xerC gene product, a member of the lambda integrase family of site-specific recombinases. *J. Bacteriol.* 1990;**172**:6973–80. doi: 10.1128/jb.172.12.6973-6980.1990.

39. Blakely G, May G, McCulloch R *et al.* Two related recombinases are required for site-specific recombination at dif and cer in E. coli K12. *Cell.* 1993;**75**:351–61. doi: 10.1016/0092-8674(93)80076-Q.
